# Trap spaces of multi-valued networks: definition, computation, and applications

**DOI:** 10.1093/bioinformatics/btad262

**Published:** 2023-06-30

**Authors:** Van-Giang Trinh, Belaid Benhamou, Thomas Henzinger, Samuel Pastva

**Affiliations:** LIS, Aix-Marseille University, Marseille 13397, France; LIS, Aix-Marseille University, Marseille 13397, France; Institute of Science and Technology, Klosterneuburg 3400, Austria; Institute of Science and Technology, Klosterneuburg 3400, Austria

## Abstract

**Motivation:**

Boolean networks are simple but efficient mathematical formalism for modelling complex biological systems. However, having only two levels of activation is sometimes not enough to fully capture the dynamics of real-world biological systems. Hence, the need for multi-valued networks (MVNs), a generalization of Boolean networks. Despite the importance of MVNs for modelling biological systems, only limited progress has been made on developing theories, analysis methods, and tools that can support them. In particular, the recent use of trap spaces in Boolean networks made a great impact on the field of systems biology, but there has been no similar concept defined and studied for MVNs to date.

**Results:**

In this work, we generalize the concept of trap spaces in Boolean networks to that in MVNs. We then develop the theory and the analysis methods for trap spaces in MVNs. In particular, we implement all proposed methods in a Python package called trapmvn. Not only showing the applicability of our approach via a realistic case study, we also evaluate the time efficiency of the method on a large collection of real-world models. The experimental results confirm the time efficiency, which we believe enables more accurate analysis on larger and more complex multi-valued models.

**Availability and implementation:**

Source code and data are freely available at https://github.com/giang-trinh/trap-mvn.

## 1 Introduction

Boolean networks are simple but efficient mathematical formalism for modelling, analysing, and controlling complex biological systems (Schwab et al. 2020). Beyond systems biology, they have been widely applied in various areas from science to engineering (Schwab et al. 2020). Boolean network models of biological systems represent genes (or other species) as nodes that can take Boolean values: 1 (active) and 0 (inactive). However, having only two levels of activation may not be enough to fully capture the dynamics of real-world biological systems (Schaub et al. 2007). There are many examples (Schaub et al. 2007; Didier et al. 2011; Mushthofa et al. 2018) where the dynamics of the system can only be modelled by considering more than two activation levels. Hence, there is a crucial need to study multi-valued networks (MVNs), which are a generalization of Boolean networks (Naldi et al. 2007; Schaub et al. 2007).

### 1.1 Related work

Despite the importance of MVNs, only limited progress has been made on developing theories, analysis methods, and tools that can support them (Mushthofa et al. 2018). First, besides simulation, the analysis of logical models is mostly based around ‘attractor’ computation, since those correspond roughly to observable biological phenotypes (Schwab et al. 2020). For example, in gene regulatory and signalling networks, attractors can correspond to cell types, cell fates, and cyclic behaviour (e.g. circadian rhythms and cell cycles). Hence, analysis of attractors could provide new insights into systems biology. However, finding all attractors of a logical model (even for the Boolean case) is challenging due to the complex dynamics of models (Schwab et al. 2020). The recent study of trap spaces of Boolean networks (Klarner et al. 2017) made a real breakthrough in the field of systems biology, as minimal trap spaces provide very good approximations of attractors and are much easier to compute. However, there has been no similar concept defined and studied for MVNs to date.

Furthermore, other biological properties, such as the gradual commitment of a cell to a specific phenotype, can be revealed through the lens of ‘succession diagrams’ constructed from the networks’ ‘maximal’ trap spaces (Rozum et al. 2021, 2022). As such, minimal trap spaces are not the only relevant form of a trap space. In the Supplementary Data (Section S4.2), we give a more detailed discussion of trap space applications in biological modelling.

Second, most of the existing studies (see, e.g. Naldi et al. 2007; Schaub et al. 2007; Didier et al. 2011 ) focus on ‘unitary’ MVNs, with only very few studies focusing on ‘general’ MVNs (see, e.g. Mushthofa et al. 2018). Third, to the best of our knowledge, very few methods/tools [see, e.g. GINsim (Naldi et al. 2007), BMA (Benque et al. 2012)] have been developed for MVNs. Most analysis methods/tools for logical models are designed for Boolean networks only [see, e.g. GINsim (Naldi et al. 2007), PyBoolNet (Klarner et al. 2017), mpbn (Paulevé et al. 2020), and Trappist (Trinh et al. 2022)].

One notable issue is that the current supporting methods for MVNs cannot handle large and complex models (Naldi et al. 2007 ; Mushthofa et al. 2018). This issue also prevents the modellers from building such models (Naldi et al. 2007), which could provide more accurate insights. Finally, a popular research direction is to convert an MVN to a Boolean network with similar dynamical behaviour, then applying the rich set of analysis methods/tools designed for Boolean networks. However, the existing Boolean encoding methods (e.g. the Van Ham Boolean mapping and its variants; Ham 1979; Didier et al. 2011) may not cover the full set of dynamics of the original MVN (Didier et al. 2011), and the encoding may even hinder the efficiency of the Boolean network methods/tools (Mushthofa et al. 2018). It is also worth noting that all mentioned encoding methods support only unitary MVNs. We believe that it is possible to develop direct and efficient methods for MVNs.

### 1.2 Our contributions

In this work, we study both general and unitary MVNs. Note that this inclusion of general networks is important not only in theory, but also in many biological applications (see Supplementary Section S1). First, we generalize the concept of trap spaces in Boolean networks to that in MVNs. Second, we prove several properties of trap spaces in MVNs including (i) the separation of minimal trap spaces, (ii) their relation with respect to the Van Ham Boolean mapping, and (iii) the characterization of trap spaces through Petri net (PN) siphons. Based on this characterization, we propose a new method utilizing answer set programming (ASP; Gebser et al. 2011) for computing different types of trap spaces of MVNs, including generic trap spaces, maximal trap spaces, minimal trap spaces, and fixed-points (a special sub-type of trap spaces). For fixed-points, we also consider another new method, which relies on the characterization of deadlocks of the PN encoding (Liu and Barkaoui 2016). We evaluate the method on a collection of real-world MVNs and show its applicability in treatment prediction on a case study of Myc-deregulation in breast cancer.

### 1.3 Paper outline

This article is structured as follows: In Section 2, we formalize the notion of trap spaces in MVNs and their relationship to the PN siphons. We propose a method based on answer-set programming for computing such trap spaces efficiently. In Section 3, we present a Python package trapmvn that implements this method for both SBML-qual and BMA models. We demonstrate the practical performance of the method on a wide selection of real-world models. We then use trapmvn as part of a case study to identify viable intervention targets in a model of Myc-associated deregulation in breast cancer. Finally, Section 4 discusses the scalability of the method, its future prospects, and highlights the role of trap spaces in reliable long-term behavioural analysis of logical models.

This article also has an associated supplement, in which we include the non-essential technical details of our methodology, as well as the full results of our benchmarks and case study. This article refers to this supplement where appropriate.

## 2 Methods

In this section, we define trap spaces of MVNs, discuss some of their theoretical properties, and finally present a method based on ASP for computing trap spaces of symbolically represented MVNs.

### 2.1 MVNs

Definition 1. *An MVN is a triple* M=(V,K,F)*such that:*

-
$V=\{v_{1},\ldots ,v_{n}\}$ *is an indexed set of nodes (variables).*
-
$K=\{K_{1},\ldots ,K_{n}\}$ *is an indexed set of integer intervals, representing the domains of variables* $v_{i}\in V$.
-
$F=\{f_{1},\ldots ,f_{n}\}$ *is an indexed set of update functions of variables* $v_{i}\in V$*. Each f_i_ has a signature* $f_{i}:\Pi_{j=1}^{n}K_{j}\to K_{i}$.

With a slight abuse of notation, we write *v_i_* to denote both the network node and the associated integer variable. Furthermore, we can also write $K_{v_{i}}$ and $f_{v_{i}}$ to denote the domain and update function of *v_i_*, respectively. We define $x\in \prod_{j=1}^{n}K_{j}$ as the ‘state’ of the MVN, with *x_i_* (or $x_{v_{i}}$) denoting the value of *v_i_* in the state *x*. We can also write x as a vector $\left[x_{1},\ldots ,x_{n}\right]$. We then write $S_{M}=\prod_{j=1}^{n}K_{j}$ to denote the set of all states (‘state space’) of network M. Finally, note that there are different possible formulations of update functions *F*, including fuzzy logic (Mushthofa et al. 2018), rule-based descriptions (Naldi et al. 2007; Delaplace and Ivanov 2020), or arithmetic expressions (Benque et al. 2012). We will return to this aspect when we discuss the encoding and manipulation of networks by our method.

The dynamics of an MVN are studied through its ‘state-transition graph’, $STG_{M}=(S_{M},\to )$. In particular, a ‘trap’ set of $STG_{M}$ is a set of states that is closed with respect to →. A trap set is called an ‘attractor’ if it is minimal, i.e. there is no other trap set that is a proper subset of this set. However, there are different ways of defining $STG_{M}$, possibly leading to significantly different behavioural features.

First, we divide MVNs based on the variable update scheme as ‘general’ (Mushthofa et al. 2018) and ‘unitary’ (Schaub et al. 2007; Delaplace and Ivanov 2020). In the general networks, the variable changes follow the update functions exactly. Meanwhile, in the unitary networks, the value of *v_i_* can only change by ‘one level’ at a time (i.e. +1 or –1). It has been argued that unitary networks better capture the continuity of biological interactions (Schaub et al. 2007), but is not true universally (Sun et al. 2014).

Note that the stepwise notion of unitary update can be in fact encoded directly into the update functions *F*. Specifically, we define ${\hat{f}}_{v}$ to represent the update function which adheres to the chosen variable update. That is, ${\hat{f}}_{v}(x)=f_{v}(x)$ for general MVNs and ${\hat{f}}_{v}(x)=x_{v}+d$ for unitary MVNs, where *d *=* *0 if $x_{v}=f_{v}(x)$, *d *=* *1 if $x_{v}<f_{v}(x)$, and *d* = – 1 when $x_{v}>f_{v}(x)$.

The second aspect of MVN semantics is the concurrency of updates. Here, two prevalent approaches are the ‘synchronous’ (all variables update together) and ‘asynchronous’ update (exactly one variable updates non-deterministically in each step).

**Figure 1.**
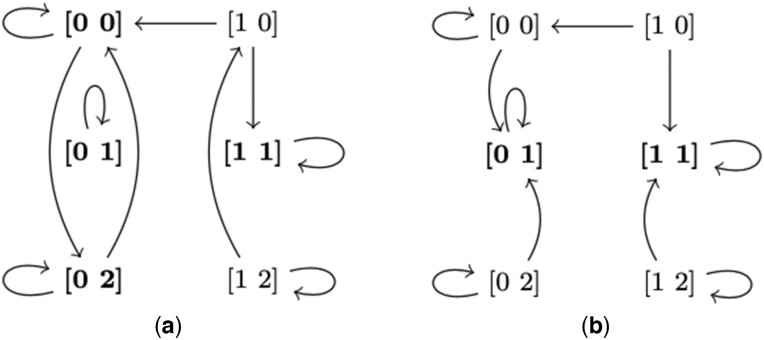
$STG_{M}$ of the example MVN under general (a) and unitary update schemes (b). Attractors are highlighted in bold.

Note that for the same MVN, different update schemes may result in vastly different state-transition graphs (Thomas 1991). Consider the MVN M with $V=\{v_{1},v_{2}\}$, $K_{1}=\{0,1\}$, and $K_{2}=\{0,1,2\}$ (Example 2 in the Supplementary Data). Figure 1(a) shows the state transition graph of M under the general update scheme. This graph has six trap sets: {[0 1]}, {[1 1]}, {[0 0], [0 2]}, {[0 1], [1 1]}, {[0 0], [0 1], [0 2]}, and {[0 0], [0 1], [0 2], [1 0], [1 1], [1 2]}. Out of these, three are attractors (minimal trap sets): {[0 1]}, {[1 1]}, and {[0 0], [0 2]}. Meanwhile, Fig. 1(b) shows the state transition graph of M using the unitary update scheme, which admits only two attractors: {[0 1]} and {[1 1]}.

### 2.2 Trap spaces of MVNs

Overall, this variability under different update schemes motivates the study of ‘trap spaces’. In the Boolean case, trap spaces have been shown to be a good approximation of network attractors, regardless of the chosen update concurrency (Paulevé et al. 2020). Here, we thus establish trap spaces for MVNs with a similar goal in mind:

Definition 2. *A space m of* $S_{M}$*is a mapping which assigns each* $v_{i}\in V$*a non-empty subset of K_i_:* $m(v_{i})\subseteq K_{i}$*. We write* $S_{M}^{⋆}$*to denote the set of all spaces of* M.

With a slight abuse of notation, we can also interpret *m* as a subset of $S_{M}$, writing x∈m for a state $x\in S_{M}$ when $x_{v}\in m(v)$ for all v∈V. Subsequently, we can define trap spaces as follows:

Definition 3. *A trap space is a space m such that for every state* x∈m*and variable* v∈V*, we have* ${\hat{f}}_{v}(x_{v})\in m(v)$.

In other words, in all states represented by *m*, all update functions only produce values that stay within *m*, meaning no $STG_{M}$ (regardless of the update concurrency) can contain a transition which leaves *m*. Consequently, trap spaces of an MVN are independent on its concurrency update, is not true for the case of attractors. Note the use of ${\hat{f}}_{v}$, meaning that the notion of trap space respects the choice of unitary or general variable update.

When interpreting spaces $m\in S_{M}^{⋆}$ as subsets of $S_{M}$, we can trivially establish a sub-space relation on $S_{M}^{⋆}$, which allows us to reason about ‘minimal’ and ‘maximal’ trap spaces:

Definition 4. *A trap space m is minimal if and only if there is no trap space* $m\prime \in S_{M}^{⋆}$*s.t.* m′⊂m*. Analogously, a trap space m is maximal if and only if* $m\subset S_{M}$*, and there is no trap space* $m\prime \in S_{M}^{⋆}$*s.t.* m⊂m′.

Note that for the notion of maximal subspace, we require that $m\subset S_{M}$. Otherwise, a trivial space $m^{⋆}$ which represents all network states (i.e. $m^{⋆}(v)=K_{v}$) is always a trap space, and always a superset of all other trap spaces.

For illustration, reconsider the example MVN (Fig. 1). Its general counterpart has six trap spaces as follows:


$$\begin{array}{c} m_{1}=\{0\}\{1\},m_{2}=\{1\}\{1\},m_{3}=\{0\}\{0,2\},\\ m_{4}=\{0,1\}\{1\},m_{5}=\{0\}\{0,1,2\},m_{6}=\{0,1\}\{0,1,2\}. \end{array}$$

Out of these, three are minimal trap spaces ($m_{1}$, $m_{2}$, and $m_{3}$) and two are maximal trap spaces ($m_{4}$ and $m_{5}$). Meanwhile, its unitary counterpart has two minimal trap spaces ({0}{1} and {1}{1}) and three maximal trap spaces ({0,1}{0,1}, {0,1}{1,2}, and {0}{0,1,2}). Finally, let us observe the following:

Theorem 1 (Separation of minimal trap spaces). *Let m_1_ and m_2_ be two distinct minimal trap spaces of* M*. Then* $m_{1}\cap m_{2}=\emptyset$.

The proof is available in the Supplementary Data as Theorem 3.

By Definition 3 and the definition of a trap set, a trap space is also a trap set of an MVN for any chosen update concurrency. As a consequence, each trap space must contain at least one attractor of the MVN. By Theorem 1, any two distinct minimal trap spaces are disjoint; thus, the attractors contained in them are also disjoint. Hence, we can conclude that regardless of the chosen update concurrency, minimal trap spaces can be used as approximations of attractors in MVNs: The number of minimal trap spaces under-approximates the number of attractors (there can be other attractors that are not contained in minimal trap spaces), and each minimal trap space over-approximates at least a single attractor. Supplementary Section S3.3 presents additional examples of relations between attractors and minimal trap spaces.

### 2.3 Translation to Boolean networks

When $K_{i}=\{0,1\}$ for all $v_{i}\in V$, we can refer to M as a ‘Boolean network’ N=(V,F) instead. For Boolean networks, the notion of trap spaces is well known, including efficient tools for computation of minimal and maximal trap spaces (Klarner et al. 2017; Paulevé et al. 2020; Trinh et al. 2022).

There are several works (Didier et al. 2011; Delaplace and Ivanov 2020) attempting to encode the multi-valued dynamics of an MVN into a Boolean network. Probably, the most widely used being the Van Ham encoding (Ham 1979), implemented in the tools GINsim (Chaouiya et al. 2011a, 2011b) and bioLQM (Chaouiya et al. 2013). The core idea of the Van Ham encoding is to expand each multi-valued variable v∈V into $|K_{v}|-1$ Boolean variables, such that an integer value *v *=* k* is encoded as the truth value of the first *k* Boolean variables. The main advantage of this encoding is that any unitary state change only involves a single Boolean variable. The disadvantage is that the encoding admits invalid states which do not correctly encode any integer and may interfere with the actual dynamics of the model. More details about the Van Ham encoding are given in the Supplementary Section S2.3.

It is known that the Van Ham encoding preserves attractors of unitary networks (Didier et al. 2011). Nevertheless, it is unclear whether this mapping also preserves trap spaces in general. To answer this question, we present the following proposition, the proofs for which can be found in the Supplementary Data.

Proposition 1. *Let* $N_{M}$*denote the Van Ham encoding of a* MVN M*. Then, it holds that:*

- *There is a general MVN* M*for which neither the maximal nor minimal trap spaces of* $N_{M}$*correspond to the respective trap spaces of* M.
- *There is a unitary MVN* M*such that the maximal trap spaces of* $N_{M}$*do not correspond to the maximal trap spaces of* M.
- *For every unitary MVN* M*, the minimal trap spaces of* $N_{M}$*correspond to the minimal trap spaces of* M.

This proposition states that the Van Ham encoding is still applicable for computation of minimal trap spaces of unitary networks, but cannot be used beyond this particular problem class.

### 2.4 Computing trap spaces through PN siphons

To tackle the problem of trap space computation of MVNs, we instead propose a different approach, based on an encoding of the problem into an answer-set programming query.

#### 2.4.1 PN encoding of MVNs

First, let us establish a characterization of trap spaces through conflict-free PN siphons.

Definition 5. *A one-safe PN is a bipartite-directed graph* P=(P,T,W)*, where P and T are disjoint finite sets of vertices called places and transitions*, *respectively. Set W describes the arcs between places and transitions:* W⊆(P×T)∪(T×P).

A *marking M* of a one-safe PN is a subset M⊆P.

For x∈P∪T, we write pred(*x*) and succ(*x*) to denote the predecessors and successors of *x* with respect to *W* (this notation naturally extends to subsets of P∪T). The dynamics of a PN are dictated by the ‘firing of transitions’, such that a transition t∈T can be fired non-deterministically in the marking *M* if pred(t)⊆M. The result is a new marking M′=(M∖pred(t))∪succ(t). This process defines a state-transition graph with vertices as the possible markings.

In the appendix of Chatain et al. (2014), the authors present a PN encoding of the ‘asynchronous’ MVN dynamics, which we briefly recall here. Note that this is not the only PN encoding of MVNs (see, e.g. Chaouiya et al. 2004, 2011a, 2011b), but to the best of our knowledge, the other encodings are not particularly suitable for the characterization of trap spaces.

Let $P_{M}$ denote the one-safe PN encoding of an MVN M based on Chatain et al. (2014). The places *P* of $P_{M}$ contain one place for every level of every MVN variable: $P=\underset{v\in V}{\cup }\left\{p_{v=i}|i\in K_{v}\right\}$. The set of transitions and arcs is then constructed such that the firing of a transition mirrors a possible update of an MVN variable. For example, let u,v∈V be such that for every $x\in S_{M}$, we have $(x_{v}=k\wedge x_{u}=l)\Rightarrow {\hat{f}}_{v}(x)=o$ (with o=k). We then create a transition *t* with $\text{pred}(t)=\{p_{v=k},p_{u=l}\}$ and $\text{succ}(t)=\{p_{v=o},p_{u=l}\}$. Such transition ‘moves’ a token from $p_{v=k}$ to $p_{v=o}$ under the assumption *u* = *l*. Note that there are many possible combinations of sets *T* and *W* that are valid for a particular M. Later, we show how we compute these sets in our case.

Finally, let $m\in S_{M}^{⋆}$ be a space of M. We write that a set of places M[m]⊆P is the ‘mirror’ of *m* when for every v∈V and i∈Kv, i∈m(v)⇔pv=i∈S. That is, M[m] contains the places corresponding to the ‘inverse’ of *m*. Observe that a state $x\in S_{M}$ is also a (trivial) space, and hence has a mirror M[x].

#### 2.4.2 Siphon characterization of MVN trap spaces

Definition 6. *A PN siphon* S⊆P*is a set of places such that for all* t∈T, (S∩succ(t))=∅*implies*(S∩pred(t))=∅.

Intuitively, a *siphon S* preserves the condition that if all places in *S* are unmarked (M∩S=∅), the siphon remains unmarked. Furthermore, for a PN $P_{M}$, we say that a siphon is ‘conflict-free’ when for all v∈V, we have that {pv=i|i∈Kv}∖S=∅. That is, a conflict-free siphon must not contain ‘all’ places encoding a particular variable. Intuitively, every conflict-free siphon of $P_{M}$ represents a mirror of ‘some’ space $m\in S_{M}^{⋆}$ (however, not all mirrors of spaces are siphons).

With this knowledge, we can observe the following theorem:

Theorem 2. *Let* M*be an MVN and* $P_{M}$*its PN encoding. Then a space* $m\in S_{M}^{⋆}$*is a trap space if and only if its mirror* M[m]*is a conflict-free siphon of* $P_{M}$.

The proof is available in the Supplementary Data as Theorem 5.

Finally, note that we can define a partial order on siphons based on the subset relation, just as we did for trap spaces. Based on the definition of a mirror and the correspondence we just proved, we can easily deduce the following (see the proofs of Theorems 6 and 7 in the Supplementary Data):

Proposition 2. *A trap space m of an MVN* M*is minimal if* M[m]*is a maximal conflict-free siphon of* $P_{M}$*. A trap space m is maximal if* M[m]*is a minimal conflict-free siphon.*

Observe that there are existing methods based on ASP (Trinh et al. 2022) and SAT solving (Nabli et al., 2016) that can handle enumeration of maximal (respectively, minimal) siphons and have been successfully used on Boolean networks before (Trinh et al. 2022). Our result shows that these methods should be also applicable to MVNs. Specifically, we aim to extend the ASP encoding from (Trinh et al. 2022) in order to support both maximal and minimal trap spaces of both general and unitary MVNs.

#### 2.4.3 Siphon computation through ASP encoding

For now, we neglected the question of update function representation for MVNs, as this depends strongly on the chosen input format. In our work, we consider both SBML-qual (Chaouiya et al. 2013) and BMA (BioModelAnalyzer) (Benque et al. 2012) formats. These differ significantly in terms of both format and capabilities.

Ultimately, we represent an update function *f_i_* using a series of ‘binary decision diagrams’ (BDDs) (Bryant 1986) $B_{1},\ldots ,B_{k}$. The network variables are encoded into Boolean variables $\underset{v\in V}{\cup }\left\{p_{v=i}|i\in K_{v}\right\}$, just as in the case of the PN encoding. Subsequently, each BDD in the series gives the necessary and sufficient conditions for achieving a particular result level:


$$f_{i}(x)=\{\begin{array}{cc} y_{1} & B_{1}(x)=1\\ \ldots & \\ y_{k} & B_{k}(x)=1 \end{array}$$

Here, $K_{i}=[y_{1},\ldots ,y_{k}]$ and *B_i_* are individual BDDs, such that each BDD can be interpreted as a Boolean function on the encoded state *x*. We also require that for each state, there is exactly one BDD *B_j_* s.t. $B_{j}(x)=1$, hence the whole function is well defined. Also, note that such *f_i_* can be easily transformed into ${\hat{f}}_{i}$, respecting either general or unitary variable update as desired.

This representation is relatively straightforward to obtain for SBML-qual models, as each update function is given as a list of Boolean terms over standard equality/inequality integer propositions. In this representation, the output values can repeat across multiple terms, and the function is not required to be exhaustive (there is a ‘default’ output value assigned to remaining inputs). However, this is easy to amend once each term is transformed into a symbolic BDD representation (see Supplementary Data).

For BMA, the translation process is more involved, as update functions are described through a language of algebraic expressions including addition (+), subtraction (–), multiplication (·), and division (/), as well as other special functions like average and rounding. Furthermore, BMA employs a normalizing transformation on function inputs when the input domain differs from the output domain. That is, an input variable *v* in the range $K_{v}=[x,y]$ is normalized to the range $K_{u}=[a,b]$ when used in the update function *f_u_*. As such, while BMA only admits integer variables, the update functions are more akin to rational functions.

While there are frameworks which partially support symbolic evaluation of such functions, e.g. algebraic decision diagrams (Bahar et al. 1997), we are not aware of any implementation that would support all the operations required by BMA. We thus opted to enumerate the whole function table and re-encode it back into individual BDDs.

Finally, to encode the dynamics of $\hat{f}$ into a PN, we consider all variable updates (v=l)→(v=k) (with *k* being l±1 when M is unitary), and then enumerate all satisfying partial valuations *w* of the BDD $p_{v=l}\wedge {\hat{B}}_{k}$ (${\hat{B}}_{k}$ being the BDD of $\hat{f}$ for output level *k*). For every valuation *w*, a transition t∈T is created which moves the token from $p_{v=l}$ to $p_{v=k}$ while ensuring that for every other relevant u∈V, place $p_{u=w(u)}$ contains a token.

Note that the number of satisfying partial valuations *w* of a BDD (and transitively, the number of PN transitions) depends on the ‘ordering’ of Boolean variables within the BDD. However, computing the optimal variable ordering is a known non-trivial problem. To reduce the number of transitions in the resulting PN, we always test *k* randomized orderings for every update function and pick the one which produces the most compact PN.

Once the PN encoding $P_{M}$ is completed, the method proceeds based on the ASP encoding of the PN siphon problem proposed by Trinh et al. (2022). This encoding produces a query which can be processed by an ASP solver such as clingo (Gebser et al. 2011), enumerating all maximal/minimal siphons of $P_{M}$. Technical aspects of this process are given in the Supplementary Data.

## 3 Results

We now present the results of our computational experiments. First, we establish the benchmark model dataset and describe the overall implementation of our tool trapmvn. We then present the performance evaluation of trapmvn in relation to the benchmark models and other tools (where applicable). Finally, we use the trap spaces arising under a wide range of therapeutic interventions in a Myc-deregulation model of breast cancer to assess the viability and reliability of such interventions.

### 3.1 Experiment setup

In our testing, we utilize SBML models from the BBM benchmark (https://github.com/sybila/biodivine-boolean-models/; note that BBM primarily publishes Booleanized SBML models, but the original multi-valued SBML files are also available.), which includes models from the GINsim tool (Chaouiya et al. 2011a, 2011b) repository as well as other independently sourced models. Furthermore, we include models from the BMA tool (http://biomodelanalyzer.org) repository (Benque et al. 2012). Disregarding trivial cases, we are left with 26 benchmark models. To the best of our knowledge, this is a highly representative sample of multi-valued models currently available in literature. The details of each benchmark model, technical description of the performance testing, as well as full results are given in Section 6 of the Supplementary Data.

### 3.2 The trapmvn package

We implement our method as a stand-alone open-source Python package trapmvn. The package provides a basic parser for both SBML and BMA models (JSON or XML). It implements the symbolic encoding for both formats using the BDD data structure from the AEON.py package (Beneš et al. 2022). From this symbolic encoding, we build either a ‘general’ or a ‘unitary’ one-safe PN. Such PN can be then encoded into the ASP query for the respective problem class and processed by the solver clingo (Gebser et al. 2011). All these steps are available to the user through several Python classes, but can be also handled directly by a simple command line interface. Finally, we also support export of the symbolic model representation back into SBML. This allows us to convert BMA models to SBML (to the best of our knowledge, this is not supported by any other tool).

### 3.3 Performance evaluation

Due to the lack of both theory and available tools, we are not aware of any existing study of trap spaces in MVNs. Nevertheless, as we have shown, the Van Ham encoding (Ham 1979) preserves the ‘minimal’ trap spaces under unitary semantics. Tools for computing minimal trap spaces of plain Boolean networks can be thus used in this case. We use bioLQM (Chaouiya et al. 2013) to compute the Van Ham encoding when necessary.

We can also consider fixed-points as a special (simpler) class of trap spaces. We can thus also compare with tools that specialize in this type of problem. Consequently, our performance evaluation consists of three parts: ‘minimal’ and ‘maximal’ trap spaces, and ‘fixed-points’.

#### 3.3.1 Minimal trap spaces

To test the performance of trapmvn when computing minimal trap spaces, we compare its runtime to two state-of-the-art tools for trap space computation in Boolean networks, trappist (Trinh et al. 2022) and mpbn (Paulevé et al. 2020) (using the Van Ham encoding). Because the number of trap spaces can be large and the knowledge of all trap spaces is not always required, we consider two experiment settings: computing just ‘one’ and computing ‘all’ minimal trap spaces. The full results of this analysis are available in Supplementary Tables S5 and S6.

**Figure 2.**
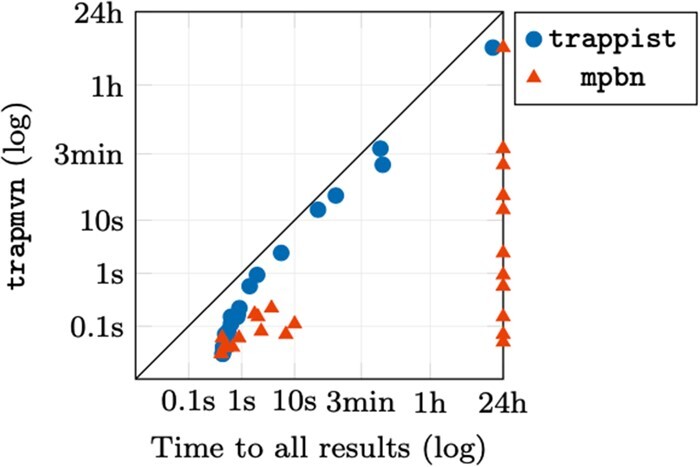
Relative performance of trapmvn compared with trappist and mpbn when counting all minimal trap spaces. The time scales are logarithmic. Points at the edge of the graph represent timeouts.

**Figure 3.**
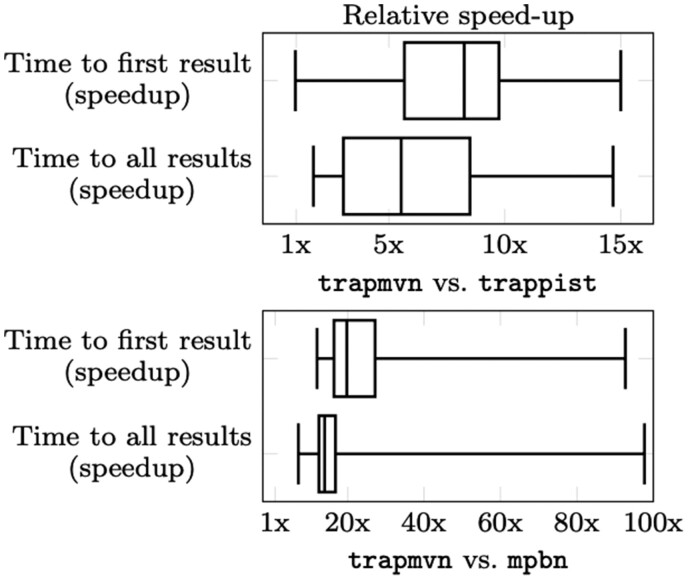
Relative speed-up in runtime of trap-mvn compared with trappist (top), res. mpbn (bottom).

In general, trapmvn can handle all models in reasonable time for both the general and unitary semantics. Focusing on the unitary case, the difference in runtime when computing all minimal trap spaces is summarized in Fig. 2. Here, we see that trapmvn performs substantially better than mpbn, and is also always faster than trappist. However, the exact speed-up is hard to assess due to the presence of logarithmic time scales. As such, we also provide a simple box plot comparing the actual speed-up in Fig. 3. Here, we see that trapmvn outperforms both methods by a significant margin in both scenarios (i.e. first result and all results). We also compute the average speed-up weighted by the absolute runtime of each benchmark (i.e. longer running benchmarks are given higher weight), showing that when computing ‘all’ trap spaces, trapmvn is 3× faster than trappist, and 58× faster than mpbn.

#### 3.3.2 Maximal trap spaces

The Van Ham encoding is not suitable for preserving ‘maximal’ trap spaces, and we are not aware of any existing method that can efficiently transform this problem to the domain of Boolean networks. As such, we cannot compare trapmvn to any other tool for this problem class. Nevertheless, we show that trapmvn can easily compute all maximal trap spaces of the considered benchmark models (Supplementary Table S7).

#### 3.3.3 Fixed-points

Finally, for the case of fixed-points, there are two viable ASP encodings, one based on PN siphons and the other based on PN deadlocks. The encoding based on deadlocks is generally more efficient for this simpler problem class. As such, we implement both variants in trapmvn and compare the results to the Booleanized results from the tools trappist (Trinh et al. 2022) and mpbn (Paulevé et al. 2020), and multi-valued results from AN-ASP (Abdallah et al. 2017). Overall, our evaluation confirms the benefits of the deadlock-based encoding, showing a 2.6× speed-up compared with the siphon encoding (Supplementary Table S8). Furthermore, we show that trapmvn also performs better than trappist or mpbn on fixed-point computation, but is outperformed by AN-ASP (1.3× speed-up), since AN-ASP is optimized solely for this specific type of problem.

### 3.4 Therapeutic interventions of Myc-deregulation

To demonstrate the practical utility of trapmvn in biological modelling, we present a case study expanding on the findings of Kreuzaler et al. (2019). The authors of Kreuzaler et al. (2019) use BMA (Benque et al. 2012) to explore viable therapeutic interventions in a large computational model of breast cancer through the lens of network attractors. Here, we show how to more reliably interpret the model by focusing on trap spaces instead.

#### 3.4.1 Modelling Myc heterogeneity in breast cancer

The Myc transcription factor is one of the key coordinators in cell proliferation and regeneration (Kortlever et al. 2017). As such, oncogenic deregulations of Myc are commonplace in many cancers, breast cancer in particular (Vita and Henriksson 2006).

Still, most tumours have been shown to consist of several genetically distinct mutants, only some of which exhibit Myc overexpression (Gerlinger et al. 2012; Heselmeyer-Haddad et al. 2012). Such heterogeneity can impede some treatments, but it can also enable new therapies that target the cooperation between the mutants (Marusyk et al. 2014; Kreuzaler et al. 2019).

In the case of Myc-related mutations, an overexpression of Myc is linked to super-competitive behaviour that causes the cancerous cells to outproliferate their healthy neighbours. However, the same overexpression is also linked to greatly increased predisposition to apoptosis. In Kreuzaler et al. (2019), the authors reveal a mechanism by which Myc^high^ mutants survive using a supply of Wnt1 transcription factor produced by a different, Myc^low^ mutant.

This process is demonstrated both experimentally on *in vivo* mouse models, as well as *in silico* on a large-scale multi-valued computational model. Based on the intervention response observed *in silico*, the authors identify a viable therapy targeting COX2 and MEK transcription factors and validate this therapy *in vivo*.

The model itself consists of 72 variables, ranging from 4 to 7 levels (MYC IN-VIVO in Supplementary Table S5). It is based on known literature, with additional validation and tuning using several independent datasets. There are five model variants: A healthy ‘wild-type’ (WT) model, Myc^low^ and Myc^high^ variants where the tumour consists homogeneously of a single mutant, and finally, mix-Myc^low^ and mix-Myc^high^ variants, which describe a heterogeneous tumour. In this case, the interaction is given as an outside assumption; there is no single model consisting of both Myc^low^ and Myc^high^ mutants sharing a state space.

To study the effects of possible therapeutic interventions, the authors compare the ‘synchronous’ attractors approximated by BMA across a range of single and dual variable knockouts. The effect of each intervention on the real-world phenotypes is assessed through variables Apoptosis and Proliferation which are directly embedded into the network. While this methodology is viable, it has shortcomings which we hope to address.

First, while BMA should be capable of computing the ‘exact’ synchronous attractors, the results in Kreuzaler et al. (2019) are only based on an ‘approximate’ method, due to the (lack of) scalability of the exact method. Second, the ‘synchronous’ update scheme can miss plausible model behaviour due to artificial synchronization between variables (Schwab et al. 2020). Meanwhile, trap spaces are universal regardless of the chosen update scheme (Paulevé et al. 2020). Finally, for non-trivial attractors, the case study in Kreuzaler et al. (2019) only considers the ‘average’ values of Apoptosis and Proliferation, which can be a poor approximation of the model’s actual admissible behaviour. This is despite the fact that BMA ‘can’ also compute the estimated attractor intervals.

#### 3.4.2 Single intervention effects

We start by replicating the single-variable knockout interventions performed in Kreuzaler et al. (2019), but in the context of trap spaces. Highlights from this analysis are presented in Table 1 (full data are available in the Supplementary Data). These results are in many aspects comparable to the original results obtained through BMA, however, they often paint a more complete picture of the model’s behaviour.

**Table 1.** Effects of selected single-node perturbations on proliferation (left) and apoptosis (right).

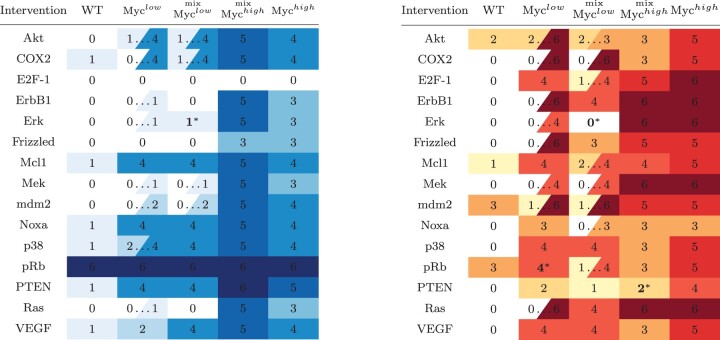

Intervals show non-trivial trap spaces. Values with asterisk show improvements over BMA approximate result.

First, there are four instances where our ‘exact’ method actually improves the precision of the original ‘approximate’ result (marked with an asterisk). Note that the more specific result is in all cases at the edge of the interval established by BMA, which means that it substantially differs from the ‘average’ considered in Kreuzaler et al. (2019).

Second, there are clearly many cases where the intervention causes the appearance of a non-trivial trap space, signified by an interval instead of a fixed value. Knowledge of these intervals is crucial when interpreting the effectiveness of interventions.

For example, consider the value of Apoptosis for the knockouts of Mcl1 and COX2 in mix-Myc^low^. The average value is the same (i.e. 3), but the admissible interval is [2,4] for Mcl1 and [0,6] for COX2. The Mcl1 intervention guarantees Apoptosis≥2, but we have no such expectation for COX2: Even though the ‘best case’ outcome is higher (i.e. 6>4), the system is not guaranteed to visit these high-value states sufficiently often to trigger apoptosis.

#### 3.4.3 Dual interventions with reliable and opportunistic effects

To systematically rank the high number of possible dual interventions, we propose to score each intervention *I* with two metrics, ‘reliability’ and ‘opportunity’, denoted by rel(*I*) and opp(*I*), respectively. The intuition is that these scores should represent the ‘worst’ and ‘best’ case scenarios (in terms of Apoptosis and Proliferation) admitted by the model for each intervention, regardless of the considered update concurrency. The exact definitions of these scores are given in Supplementary Section S5.4.

We then focus on the admissible dual-knockout interventions: both knockouts must be druggable (according to Kreuzaler et al. 2019), and their combination cannot increase Apoptosis beyond level three in the WT model. This leaves 995 interventions for which we compute both scores. For presentation purposes, we sort the interventions by the ‘average’ of these two scores.

**Figure 4.**
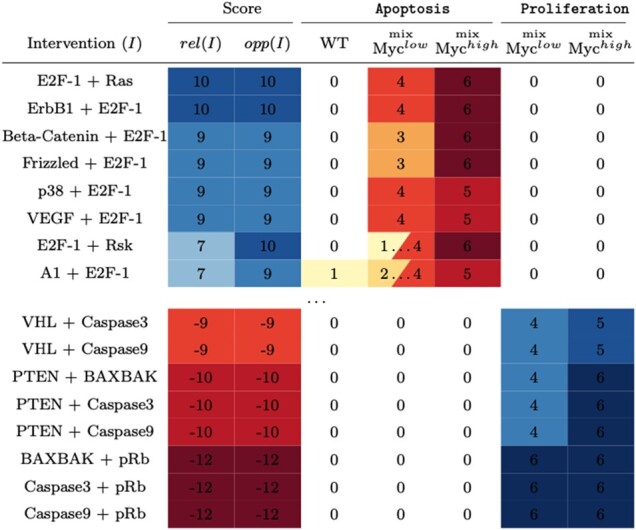
Eight best and worst dual interventions (out of 995 tested), together with their reliability and opportunity scores, as well as their effect on the relevant model variables.

The eight best and the eight worst interventions are shown in Fig. 4. As we can see, some of the best interventions admit a non-trivial trap space for the mix-Myc^low^ variant. However, even in this case, the reliability and opportunity scores are not vastly different. This raises a natural question regarding the prevalence of differences between reliability and opportunity scores in general. We further study this question in the Supplementary Data (Supplementary Section S5.4 and Supplementary Fig. S6, in particular), where we show that substantial differences between the two scores are in fact common even within the best scoring interventions.

## 4 Discussion

In this article, we formalized the notion of trap spaces in MVNs, then explored and proved properties of such trap spaces with applications in the analysis and control of MVNs. One notable property is that trap spaces of MVNs are independent to concurrency update schemes. We argued that, akin to Boolean networks, trap spaces can serve as approximation of network attractors. Furthermore, we showed that not all useful properties of trap spaces are preserved through a Boolean encoding of an MVN. For example, maximal trap spaces of unitary networks, crucial for the construction of the network’s succession diagram (Rozum et al. 2021), are not preserved. Next, we made a connection between trap spaces of an MVN and siphons of its PN encoding. Based on this relationship, we proposed and implemented a new ASP method for computing different types of trap spaces of an MVN.

### 4.1 Method performance

We have evaluated the time efficiency of our method on real-world models collected from the literature. We show that the method scales well with the network size and it can handle large-scale realistic models for both the general and unitary semantics. The indirect approach (i.e. through a Boolean encoding) is only applicable for the case of fixed-points and minimal unitary trap spaces. In these cases, the direct approach (i.e. our method) outperforms the best indirect method.

In particular, we discuss the factors that contribute to the running time of each compared method (all are ASP-based) with respect to the minimal trap space computation. Aside from the absolute number of solutions, the practical complexity of an ASP query is affected by its number of atoms and its ‘density’, i.e. the ratio between the number of ASP rules and atoms. Through a systematic analysis (details are available in Supplementary Section S6.1), we see that an increase in density is always accompanied by increased runtime. Query density is clearly not the only indicator of ASP problem complexity. However, assuming we control for other relevant factors (solution count, tool/method, update scheme, model format, etc.), query density appears to be a relevant metric for comparing the complexity of two minimal trap space computation problems.

We also analyse the runtime of our method trap-mvn in the general and unitary cases. In general, trap-mvn needs more time for the general case than for the unitary case, as the PN encoding of a general MVN has more transitions than that of its unitary counterpart. The detailed discussions are given at the end of Section S6.1.

Finally, we have tried to also compare the performance of trap-mvn to BMA. However, in its current state, we were only able to run the ‘approximate’ attractor detection method, which is (as expected), much faster than any of the tested ‘exact’ methods. There appears to be no public documentation or reproducible artefact concerning the exact solver-based approach for the currently available version of BMA. However, the authors of Kreuzaler et al. (2019) note that the exact method was not able to efficiently analyse the Myc heterogeneity model. As such, it seems unlikely that it would provide competitive performance on the remaining models in our benchmark set.

### 4.2 Reliable identification of network interventions

Subsequently, we studied the practical applicability of our method on a model of Myc-deregulation from Kreuzaler et al. (2019). First, we indeed found instances where our exact method can improve the approximate results obtained in Kreuzaler et al. (2019). Furthermore, as we show in Table 1, the behaviour of the model can often admit significant fluctuations and uncertainty. These are not reflected in the average value within attractor states, as considered in Kreuzaler et al. (2019). This highlights the need for rigorous and exhaustive analysis of such behaviour uncertainty.

With this goal in mind, we propose a ‘reliability’ and ‘opportunity’ score to assess the viability of therapeutic interventions. Based on these scores, we see that even interventions that introduce some amount of uncertainty can be safely considered among the most viable (Fig. 4). Furthermore, we study how prevalent is the variability of intervention scores in our dataset. As shown in Supplementary Fig. S6, for almost half of the interventions (439/995), the difference is zero. However, for 463/995 interventions, the model still admits a difference of three or more points. Such difference can impact the expected observed biological phenotypes if not taken into account. Furthermore, our results reveal that even though the highest score variability is associated with scores we would not consider particularly viable, very viable interventions (e.g. average score >5) can still exhibit high variability (≤6).

Notably, the COX2 + MEK intervention chosen in Kreuzaler et al. (2019) has a reliability and opportunity score equal to 5, placing it on rows 65–149 of the table in Fig. 4 (there are 84 interventions with the same scores). While this is not among the best scores, it is still better than 85%–93% of interventions, meaning we retained the viability of this particular intervention. Furthermore, it should be noted that our screening for ‘viable’ interventions is rather rudimentary: in practice, some of the top perturbations might be ruled out due to factors other than the WT Apoptosis result (e.g. other side effects not captured by this model).

Overall, these results support the claim that logical models can exhibit a high level of variability in their outcomes and it is crucial to take this variability into account when drawing conclusions. Due to their favourable computational and theoretical properties, trap spaces appear to be uniquely suited for this task.

### 4.3 Applications and future prospects

Observe that the results we explored in this article can be also useful for other types of MVN analysis. First, there is a trap space-based model reduction technique. This relies on the fact that, given a single trap space, we can obtain a simpler MVN that captures the self-contained dynamics of the states within this trap space. Second, the set of minimal trap spaces of an MVN can be seen as an approximation of its attractors, regardless of the update concurrency. Third, there are existing control methods for Boolean networks based on trap spaces (Fontanals et al. 2020; Rozum et al. 2022). It should be possible to extend these methods to control of MVNs. We discuss the details of these applications in Section S3.3.

As a perspective, we also plan to attack the attractor detection problem for MVNs, as trap spaces only capture the network’s static behaviour, whereas attractors can also capture its more complex dynamical aspects. Exploiting the relation between attractors and trap spaces of MVNs is a potentially promising direction for this problem. Furthermore, we plan to develop direct and efficient control methods for MVNs, because the control problem is crucial in systems biology. Arguably, it can be seen as ‘a sequel’ of the trap space or attractor analysis (Fontanals et al. 2020).

## Supplementary Material

btad262_Supplementary_Data

## Data Availability

Source code and data are freely available at https://github.com/giang-trinh/trap-mvn.
