# Supplementary material for: Trap spaces of multi-valued networks: definition, computation, and applications
Source: Bioinformatics. 2023 Jun 30;39(Suppl 1):i513–22. doi: 10.1093/bioinformatics/btad262 (PMC10311308; doi:10.1093/bioinformatics/btad262)
Supplement: btad262_Supplementary_Data [file btad262_supplementary_data.pdf]

Trap spaces of multi-valued networks: Definition, computation, and applications (Supplementary Material)

Abstract. This supplementary material expands on the contents of the main paper. The structure of the supplement roughly follows the structure of the main paper, giving additional technical details wherever applicable. Note that some notation has been updated compared to the main paper. This is mainly to ease readability and reduce ambiguity in the absence of a strict page limit. Additionally, we include multiple illustrative examples which also could not be included in the main paper due to space restrictions.

1 Introduction

Boolean networks are simple but efficient mathematical formalism for modeling, analyzing, and controlling complex biological systems [49,45]. Beyond systems biology, they have been widely applied to various areas from science to engineering [58,49]. Boolean network models of biological systems represent genes as nodes that can take Boolean values: 1 (active) and 0 (inactive). However, having only two levels of activation is sometimes not always enough to fully capture the dynamics of real-world biological systems [47,38]. There are many examples [22,15,38,4] from the literature where the dynamics of the system can only be modeled by considering more than two activation levels. One classic example is the lac operon regulatory system [34] whose key attractors cannot be characterized using a Boolean encoding. Some others are multi-state models [53], strictly ternary models [11], and models employing a combination of Boolean and ternary variables [43]. Hence, there is a crucial need of multi-valued networks that are a generalization of Boolean networks [47,40].

Despite the importance of multi-valued networks for modeling biological systems, only limited progress has been made on developing theories, analysis methods, and tools that can support them [38]. First, besides simulation, the analysis of logical models is mostly based on attractor computation, since those correspond roughly to observable biological phenotypes [49]. The recent use of trap spaces in Boolean networks [29] made a real breakthrough in the field of systems biology as minimal trap spaces provide very good approximations for attractors and it is much easier to compute them. However, there has been no similar concept defined and studied for multi-valued networks to date. Second, most of the existing studies (see, e.g., [47,40,15,17,14]) focus on *unitary* multi-valued networks, only very few studies focus on *general* multi-valued networks (see, e.g., [44,38]). Third, to the best of our knowledge, very few methods/tools (see,

e.g., `GINsim` [40], `BMA` [5]) have been developed for multi-valued networks, most of analysis methods/tools for logical models are designed for Boolean networks (see, e.g., `GINsim` [40], `PyBoolNet` [30], `mpbn` [42], `Trappist` [55]). Moreover, these methods/tools also focus mainly on unitary multi-valued networks. One notable issue is that the current supporting methods for multi-valued networks cannot handle large and complex models [40,38]. This issue also prevents the modellers to build such models [40], which can provide more accurate insights. Finally, one popular research direction is to convert a multi-valued network to a Boolean network with the same dynamical behaviour, then we can apply the rich set of analysis methods/tools designed for Boolean networks. However, the existing Boolean encoding methods (e.g., the Van Ham Boolean mapping and their variants [26,15,17,14]) may not cover the full set of dynamics of the original multi-valued network on the one hand [17] and the encoded Boolean network may hinder the efficiency of the Boolean network methods/tools on the other hand [38]. It is also worth noting that all the above Boolean encoding methods support only unitary multi-valued networks. We believe that it is possible to develop direct but efficient methods for multi-valued networks.

In this work we first generalize the concept of trap spaces in Boolean networks to that in multi-valued networks. Second, we prove several properties of trap spaces in multi-valued networks including a) the characterization of trap spaces, b) the separation of minimal trap spaces, and c) the relations with respect to the Van Ham Boolean mapping. Third, we show the theoretical applications of trap spaces in the analysis and control of multi-valued networks. Next, we make a connection between trap spaces of a multi-valued network and siphons of its Petri net encoding. From this connection, we propose a new method based on answer set programming [23] for computing different types of trap spaces of a multi-valued network including generic trap spaces, maximal trap spaces, minimal trap spaces, and fixed points (special trap spaces). In particular, we also propose another new method for the case of fixed points, which relies on the characterization of deadlocks of the Petri net encoding [33]. After showing the applicability of our methods via a realistic case study, we evaluate their performance by conducting experiments on real-world models.

Note that all the above results are applicable for both general and unitary multi-valued networks. Although unitary multi-valued networks have attracted more attention than general ones (the main reason may be the availability of Boolean mapping methods for unitary multi-valued networks), the choice between the unitary and general semantics is still not conclusive and depends on the timescale assumptions on the modeled variables. In the unitary semantics, every gradual value change is always “observable” by the system. Nevertheless, we could imagine a biochemical process where the differences in timescales between variables [48] lead to a value change that is not visible to the downstream variables (i.e., it happens too quickly). Indeed, in most cases, the unitary semantics is the “safer” assumption, but by supporting both cases, the modelers can choose appropriate updating scheme for their case. It is worth noting that there are some work [51,60] using general multi-valued networks to model and analyze

real biological systems with many new insights are obtained. `CellNetAnalyzer`[1], a famous toolbox for exploring structural and functional properties of metabolic, signaling, and regulatory networks, supports general rather than unitary multi-valued networks, although its analysis methods for general multi-valued networks mainly rely on the explicit construction of the state transition graph. In addition, any unitary multi-valued network can be equivalent in dynamics to a general multi-valued network (see Section 3.2 for the detailed conversion), but the opposite direction is not true. Hence, the consideration of general multi-valued networks can provide not only biological but also theoretical benefits.

The remainder of this supplement is structured as follows: Section 2 introduces the basic concepts including Boolean networks, multi-valued networks, Petri nets and their siphons. Section 3 presents the formal definition, properties, and theoretical applications of trap spaces in multi-valued networks. Section 4 shows the connection between trap spaces of multi-valued networks and siphons of their Petri net encoding. It also shows the details of the proposed methods for trap space computation based on the connection. Section 5 shows a case study using a realistic model from the literature. Section 6 reports the experimental results for evaluating the efficiency of the proposed methods. Finally, Section 7 concludes the paper and draws future work.

2 Preliminaries

2.1 Boolean networks

Definition 1. *A Boolean Network (BN) is a tuple $\mathcal{N} = \langle V, F \rangle$ where:*

- $V = \{v_1, \ldots, v_n\}$ *is the set of nodes. We use v_i to denote both the node v_i and its associated Boolean variable.*
- $F = \{f_1, \ldots, f_n\}$ *is the set of update functions. Each function f_i is associated with node v_i and satisfies $f_i \colon \mathbb{B}^n \mapsto \mathbb{B}$ where $\mathbb{B} = \{0, 1\}$.*

A Boolean function is *locally-monotonic* if it can be represented by a formula in disjunctive normal form in which all occurrences of any given literal are either negated or non-negated [42]. A Boolean network is said to be locally-monotonic if all its Boolean functions are locally-monotonic. Otherwise, this model is said to be non-locally-monotonic.

A state of a Boolean network is a vector $v = [v_1, \ldots, v_n]$ where $v_i \in \mathbb{B}$ represents the value of node v_i. State $v \in \mathbb{B}^n$ can be seen as a mapping $v \colon V \mapsto \mathbb{B}$ that assigns either 0 (inactive) or 1 (active) to each node. We denote the set of all possible states of a Boolean network $\mathcal{N}$ by $\mathcal{S}_{\mathcal{N}} = \mathbb{B}^n$.

[1] http://www2.mpi-magdeburg.mpg.de/projects/cna/cna.html

Update schemes of Boolean networks At each time step t, node v_i can update its state by

$$v_i(t+1) = f_i(v(t))$$

where $v(t)$ is the state of $\mathcal{N}$ at time t, $v_i(t+1)$ is the state of node v_i at time $t+1$. An update scheme of a Boolean network specifies the way that the nodes update their states through time evolution [54]. Following the update scheme, the Boolean network transits from a state to another state (possibly identical). This transition is called the *state transition* and denoted by $\rightarrow \subseteq \mathcal{S}_{\mathcal{N}} \times \mathcal{S}_{\mathcal{N}}$. Then the dynamics of $\mathcal{N}$ is captured by the directed graph $(\mathcal{S}_{\mathcal{N}}, \rightarrow)$ called the State Transition Graph (STG). There are two main types of update schemes [54]: synchronous, where all the nodes are update simultaneously, and fully asynchronous, where only one node is nondeterministically selected to be updated. We denote $(\mathcal{S}_{\mathcal{N}}, \rightleftharpoons)$ (resp. $(\mathcal{S}_{\mathcal{N}}, \hookrightarrow)$) the STG of $\mathcal{N}$ under the synchronous (resp. fully asynchronous) update scheme.

Trap sets and trap spaces of Boolean networks A non-empty set $T \subseteq \mathcal{S}_{\mathcal{N}}$ is a trap set with respect to $\rightarrow$ if for every $x \in T$ and $y \in S$ with $x \rightarrow y$ it holds that $y \in T$ [29]. An attractor A of $\mathcal{N}$ with respect to $\rightarrow$ can be defined as a minimal trap set of $(\mathcal{S}_{\mathcal{N}}, \rightarrow)$. That is there is no other trap set such that it is a proper subset of this attractor. An attractor of size 1 is called a fixed point, otherwise a cyclic attractor [29].

A sub-space m of a Boolean network $\mathcal{N} = \langle V, F \rangle$ is a mapping $m \colon V \mapsto \mathbb{B} \cup \{\star\}$. $m(v_i) \in \mathbb{B}$ means that the value of v_i is fixed in m and v_i is called a fixed variable. $m(v_i) \in \star$ means that the value of v_i is free in m and v_i is called a free variable. A sub-space m is equivalent to a set of states:

$$\mathcal{S}_{\mathcal{N}}[m] := \{s \in \mathcal{S}_{\mathcal{N}} \mid \forall v \in V \colon m(v) \in \mathbb{B} \implies s(v) = m(v)\}.$$

We denote $\mathcal{S}^{\star}_{\mathcal{N}} = (\mathbb{B} \cup \{\star\})^n$ the set of all possible sub-spaces of $\mathcal{N}$.

A sub-space is a trap space if it is also a trap set. Then we define a partial order $<$ on $\mathcal{S}^{\star}_{\mathcal{N}}$ as: $m < m'$ if and only if $\mathcal{S}_{\mathcal{N}}[m] \subset \mathcal{S}_{\mathcal{N}}[m']$. From this partial order, we can define a minimal or maximal trap space as follows. A trap space m is minimal if there is no trap space m' such that $m' < m$. A trap space m is maximal if $\mathcal{S}_{\mathcal{N}}[m] \neq \mathcal{S}_{\mathcal{N}}$ and there is no trap space m' such that $m' > m$ and $\mathcal{S}_{\mathcal{N}}[m'] \neq \mathcal{S}_{\mathcal{N}}$. It is worth noting that trap spaces of a Boolean network are independent of the update scheme [29].

For illustration, let us consider the Boolean network $\mathcal{N}$ shown in Example 1. Figures 1(a) and 1(b) show $(\mathcal{S}_{\mathcal{N}}, \rightleftharpoons)$ and $(\mathcal{S}_{\mathcal{N}}, \hookrightarrow)$, respectively. Both $(\mathcal{S}_{\mathcal{N}}, \rightleftharpoons)$ and $(\mathcal{S}_{\mathcal{N}}, \hookrightarrow)$ have only two fixed points ({[0 1 0]} and {[1 1 0]}). {[0 0 0], [0 1 0], [0 1 1]} is a trap set of both $(\mathcal{S}_{\mathcal{N}}, \rightleftharpoons)$ and $(\mathcal{S}_{\mathcal{N}}, \hookrightarrow)$. However, it is not an attractor because it is not minimal. $\mathcal{N}$ has all ten trap spaces along with their

equivalent sets of states as follows:

$$\begin{aligned}
\{[0\ 1\ 0]\} &\sim n_1 = 010,\\
\{[1\ 1\ 0]\} &\sim n_2 = 110,\\
\{[0\ 1\ 0], [1\ 1\ 0]\} &\sim n_3 = \star 10,\\
\{[1\ 1\ 0], [1\ 1\ 1]\} &\sim n_4 = 11\star,\\
\{[0\ 0\ 0], [0\ 1\ 0]\} &\sim n_5 = 0\star 0,\\
\{[0\ 1\ 0], [0\ 1\ 1]\} &\sim n_6 = 01\star,\\
\{[0\ 0\ 0], [0\ 0\ 1], [0\ 1\ 0], [0\ 1\ 1]\} &\sim n_7 = 0\star\star,\\
\{[0\ 0\ 0], [0\ 1\ 0], [1\ 0\ 0], [1\ 1\ 0]\} &\sim n_8 = \star\star 0,\\
\{[0\ 1\ 0], [0\ 1\ 1], [1\ 1\ 0], [1\ 1\ 1]\} &\sim n_9 = \star 1\star,\\
\{[0\ 0\ 0], [0\ 0\ 1], [0\ 1\ 0], [0\ 1\ 1], [1\ 0\ 0], [1\ 0\ 1], [1\ 1\ 0], [1\ 1\ 1]\} &\sim n_{10} = \star\star\star.
\end{aligned}$$

Note that n_{10} is a special trap space where all variables are free (i.e., $\mathcal{S}_{\mathcal{N}}[n_{10}] = \mathcal{S}_{\mathcal{N}}$). We can see that $\mathcal{N}$ has two minimal trap spaces (n_1 and n_2) and three maximal trap spaces (n_7, n_8, and n_9).

Example 1. Consider the following Boolean network $\mathcal{N} = \langle V, F\rangle$ where $V = \{v_1, v_2, v_3\}$ and

$$F = \begin{cases} f_1 = v_1 \wedge v_2,\\ f_2 = 1,\\ f_3 = 0.\end{cases}$$

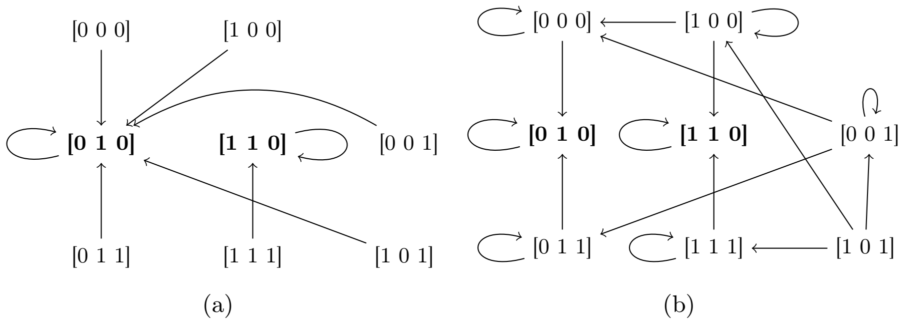

Fig. 1: The STGs under the synchronous update scheme (a) and under the fully asynchronous update scheme (b) of the Boolean network shown in Example 1. Attractors are highlighted in bold.

2.2 Multi-valued networks

Definition 2. *A Multi-Valued Network (MVN) is a tuple* $\mathcal{M} = \langle V, K, F \rangle$ *where:*

- $V = \{v_1, \dots, v_n\}$ *is the set of nodes. We use* v_i *to denote both the node* v_i *and its associated integer variable.*
- $K = \{K_1, \dots, K_n\}$ *is the set of intervals of integers. Interval* K_i *(or* K_{v_i}*) denotes the possible values of node* v_i *(i.e., the* domain *of* v_i*). Note that it is possible that* $|K_i| \neq |K_j|, i \neq j$*. In the literature, it is conventionally assumed that* $K_i = \{0, \dots, |K_i| - 1\}$*.*
- $F = \{f_1, \dots, f_n\}$ *is the set of update functions. Each function* f_i *is associated with node* v_i *and satisfies* $f_i : \prod_{j=1}^{n} K_j \mapsto K_i$*.*

To our best knowledge, there is no unified arithmetic formulation of update functions. [38] uses fuzzy logic to represent update functions, but it only deals with the case that $K_i = K_j, \forall i, j$, i.e., all the nodes have the same domain. [14] and [40] use rule-based descriptions, where each rule includes a set of conditions on input nodes of a node v_i and the value that v_i will receive under these conditions. We shall discuss the formulation of update functions of multi-valued networks in detail in Subsection 2.4.

Similar to a state of a Boolean network, we can define a state of a multi-valued network as a vector $v = [v_1, \dots, v_n]$ where $v_i \in K_i$ represents the value of node v_i. A state v can be seen as a mapping that maps every node v_i to a value in K_i. We denote the set of all possible states of a multi-valued network $\mathcal{M}$ by $\mathcal{S}_{\mathcal{M}} = \prod_{i=1}^{n} K_i$.

General and unitary multi-valued networks There are two main types of multi-valued networks: *general* [44] and *unitary* [47,14]. For general multi-valued networks, the value of node v_i is updated by

$$v_i(t+1) = f_i(v(t)),$$

where $v(t)$ is the state of the multi-valued network at time step t and $v_i(t+1)$ is the state of node v_i at time step $t+1$. For unitary multi-valued networks, the update semantics is as follows:

$$v_i(t+1) = \begin{cases} v_i(t) \text{ if } v_i(t) = f_i(v(t)), \\ v_i(t) + 1 \text{ if } v_i(t) < f_i(v(t)), \\ v_i(t) - 1 \text{ if } v_i(t) > f_i(v(t)), \end{cases}$$

for all node $v_i \in V$. Similar to the case of Boolean networks, an updating scheme is also employed for a multi-valued network. Now, the concepts of state transition graphs, trap sets and attractors for multi-valued networks are similar to those for Boolean networks.

Example 2. We use the following general multi-valued network $\mathcal{M} = \langle V, K, F \rangle$ as our straight example.

– $V = \{v_1, v_2\}$
– $K_1 = \{0,1\}$, $K_2 = \{0,1,2\}$
–

$$F = \begin{cases} f_1 = \begin{cases} 0 \text{ if } v_1 = 0 \text{ or } v_2 = 0, \\ 1 \text{ otherwise.} \end{cases} \\ f_2 = \begin{cases} 0 \text{ if } v_1 = 2 \text{ or } v_2 = 2, \\ 1 \text{ if } v_1 = 1 \text{ and } v_2 = 0 \text{ or } v_1 = 1 \text{ and } v_2 = 1 \text{ or } v_1 = 0 \text{ and } v_2 = 1, \\ 2 \text{ otherwise.} \end{cases} \end{cases}$$

Figure 2(a) shows the state transition graph of $\mathcal{M}$ under the fully asynchronous update scheme. $\mathcal{M}$ has all six trap sets: {[0 1]}, {[1 1]}, {[0 0], [0 2]}, {[0 1], [1 1]}, {[0 0], [0 1], [0 2]}, and {[0 0], [0 1], [0 2], [1 0], [1 1], [1 2]}. However, $\mathcal{M}$ has all three attractors: two fixed points ({[0 1]} and {[1 1]}) and one cyclic attractor ({[0 0], [0 2]}). Figure 2(b) shows the STG of the unitary counterpart of $\mathcal{M}$. This counterpart has only two fixed points ({[0 1]} and {[1 1]}).

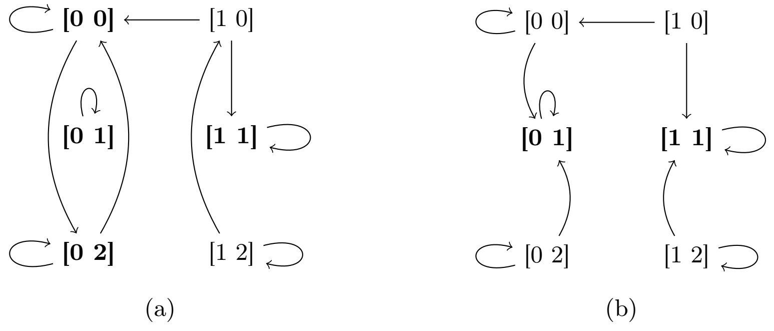

Fig. 2: The state transition graphs under the fully asynchronous update scheme of (a) the general multi-valued network shown in Example 2 and (b) its unitary counterpart. Attractors are highlighted in bold.

2.3 Van Ham Boolean mapping

In [26], an encoding of *unitary* multi-valued networks into Boolean networks is proposed. This encoding preserves many (but not all) dynamical properties of MVNs and as such, the resulting Boolean network can be often used in the place of the original MVN.

For completeness, we briefly recall the definition of the Van Ham encoding: Let us assume a multi-valued network $\mathcal{M} = \langle V, K, F \rangle$. Now, let us assume a variable $v \in V$ such that $|K_v| > 2$, i.e. variable v is not Boolean (for Boolean variables, no transformation is necessary). Without the loss of generality, we assume

K_v is an interval that starts from zero (i.e. $K_v = \{0, \ldots, |K_v| - 1\}$). For convenience, we define max_v to be $|K_v| - 1$. The Van Ham encoding expands such variable v into max_v Boolean variables which we denote $u_{v=1}, u_{v=2}, \ldots, u_{v=max_v}$.

The interpretation of these Boolean variables is the following: $v = x$ if and only if $u_{v=i} = \mathit{true}$ for all $i \leq x$ and $u_{v=j} = \mathit{false}$ for all $j > x$. For example, $v = 0$ when all the Boolean variables are *false*. Meanwhile, $v = 2$ when $u_{v=1}$ and $u_{v=2}$ are *true*, but the remaining Boolean variables are *false*.

The update functions of each variable are then altered to accept such encoded values. The specific way in which this is performed depends on the actual representation of the update functions. However, the core idea of this transformation is that any test for equality or inequality on multi-valued variable v can be transformed into a logical test on the Boolean variables $u_{v=i}$. For example, to test that $v \geq k$, we only need to check whether $u_{v=k}$ is *true*. To test that $v = k$, we have to check that $u_{v=k}$ is *true* and $u_{v=k+1}$ is *false*, and so on. Since addition, multiplication and other arithmetic operations on a final domain can be expanded into logical operations, this process can be also performed for function representations that incorporate integer arithmetic.

An important property of this encoding is that a single unitary integer transition (i.e. a $+/-1$ change) always corresponds to the update of a single Boolean variable. For example, a state change from $v = 1$ to $v = 2$ only updates Boolean variable $u_{v=2}$ (from *false* to *true*).

However, note that the encoded Boolean network also admits values that do not correspond to any valid integer interpretation of variable v (e.g. $u_{v=1} = \mathit{false}$ and $u_{v=2} = \mathit{true}$). In general, the Booleanised update functions ensure that a correctly encoded state cannot transition into an incorrectly encoded state. Hence, for example, a simulation initiated in a valid state will only visit valid states. However, the presence of these invalid states can still influence some aspects of formal dynamical analysis, such as the computation of maximal trap spaces (as we outline later in Section 3.2).

2.4 Petri nets

Definition 3. *A* Petri net *is a weighted bipartite directed graph* (P, T, W)*, where* P *is a non-empty finite set of vertices called* places*,* T *is a non-empty finite set of vertices called* transitions*,* $P \cap T = \emptyset$*, and* $W : (P \times T) \cup (T \times P) \mapsto \mathbb{N}$ *is a weight function attached to the arcs.*

A *marking* for a Petri net is a mapping $M : P \mapsto \mathbb{N}$ that assigns a number of tokens to each place. A place p is marked by a marking M if and only if $M(p) > 0$. Marking M can be seen as a subset of P that contains all marked places by M. We shall write $pred(x)$ (resp. $succ(x)$) to represent the set of vertices that have a (non-zero weighted) arc leading to (resp. coming from) x. In this work, we consider a class of Petri nets called 1-safe Petri nets where every place has at most 1 token and all arcs are of weight 1. In this case, weights are implicitly omitted in the arcs of a Petri net.

A transition $t \in T$ is *enabled* at a marking M if and only if $pred(t) \subseteq M$. The firing of t leads to a new marking M' specified by $M' = (M \backslash pred(t)) \cup succ(t)$. Note that when multiple transitions are enabled, we need to embed one firing scheme (similar to the update scheme of a multi-valued network) to the Petri net. The classical firing scheme is that only one of the enabled transition is non-deterministically chosen to fire [37].

Link between Petri nets and multi-valued networks The link between Boolean networks and Petri nets was originally established in [9]. This encoding was then extended to multi-valued networks in two ways, either in [8] with non 1-safe Petri nets or more recently in [10] with 1-safe Petri nets but many more places. Since we use 1-safe Petri nets in this work, we briefly recall the encoding by [10] here.

Informally, the encoded Petri net $\mathcal{P}$ of a multi-valued network $\mathcal{M}$ has one place per possible level of a node. Denote $p_{v_i}^j$ the place corresponding to the level j of node v_i. A transition $t_{v_i}^k$ of $\mathcal{P}$ corresponds to a condition for changing the state of node v_i. Such a condition can typically be built from the update function of the node and be in the form that v_i changes from state $j_1 \in K_i$ to state $j_2 \in K_i$ if and only if the conjunct of value constraints on the input nodes of v_i holds. Then it creates a directional arc from $t_{v_i}^k$ to $p_{v_i}^{j_2}$, a directional arc from $p_{v_i}^{j_1}$ to $t_{v_i}^k$, and bidirectional arcs from $t_{v_i}^k$ to the places corresponding to the satisfying values of the input nodes of v_i that appear in the conjunct of value constraints. Let s be a state of $\mathcal{M}$ and M_s be its corresponding marking in $\mathcal{P}$. It holds that $v_i \in V$, $s(v_i) = j$ if and only if $M_s(p_{v_i}^j) = 1$. Note also that at any marking M of $\mathcal{P}$, it always holds that

$$\sum_{j=0}^{|K_i|} M(p_{v_i}^j) = 1, \forall v_i \in V.$$

More details of this encoding can be found at Appendix A of [10].

The main property of this encoding is that it is completely faithful with respect to the update scheme of the original multi-valued network. For each node v of $\mathcal{M}$, only transitions corresponding to v can change the current marking of the places corresponding to v (i.e., $p_v^j, \forall j \in K_v$). In addition, at any marking M at most one of such transitions is enabled because $\sum_{j \in K_v} M(p_v^j) = 1$ holds. Hence, for any update scheme in $\mathcal{M}$, we have a corresponding firing scheme in $\mathcal{P}$, which preserves the equivalence between the dynamics of $\mathcal{M}$ and $\mathcal{P}$.

Note that to obtain the Petri net encoding of the unitary counterpart $\mathcal{U}$ of $\mathcal{M}$, we can only adjust $\mathcal{P}$ a bit. Specifically, let $\mathcal{P}_\mathcal{U}$ be the Petri net encoding of $\mathcal{U}$. $\mathcal{P}_\mathcal{U}$ and $\mathcal{P}$ share the same set of places. For every transition $t_{v_i}^j$ of $\mathcal{P}$, we add this transition along with all the arcs connecting to it to $\mathcal{P}_\mathcal{U}$. Let $p_{v_i}^{j_1}$ and $p_{v_i}^{j_2}$ be the input place and output place of $t_{v_i}^j$ corresponding to node v_i, respectively. If $j_2 > j_1$, we replace in $\mathcal{P}_\mathcal{U}$ the arc $(t_{v_i}^j, p_{v_i}^{j_2})$ by the arc $(t_{v_i}^j, p_{v_i}^{j_1+1})$. If $j_2 < j_1$, we replace in $\mathcal{P}_\mathcal{U}$ the arc $(t_{v_i}^j, p_{v_i}^{j_2})$ by the arc $(t_{v_i}^j, p_{v_i}^{j_1-1})$. Following the update semantics of a unitary multi-valued network, there is an equivalence between the dynamics of $\mathcal{U}$ and $\mathcal{P}_\mathcal{U}$.

For illustration, let us reconsider the general multi-valued network $\mathcal{M}$ shown in Example 2. Figure 3(a) shows the Petri net encoding of $\mathcal{M}$. Places $p^0_{v_1}$ and $p^1_{v_1}$ (resp. $p^0_{v_2}$, $p^1_{v_2}$, and $p^2_{v_2}$) represents two (resp. three) possible values of node v_1 (resp. v_2). Transition $t^1_{v_1}$ represents the change from 1 to 0 of node v_1 with the condition that the current value of v_2 is 0 and the current value of v_1 is 1. Transition $t^1_{v_2}$ represents the change from 0 to 2 of node v_2 with the condition that the current value of v_2 is 0 and the current value of v_1 is 0. Marking $M = \{p^0_{v_1}, p^2_{v_2}\}$ corresponds to state [0 2] of $\mathcal{M}$. Figure 3(b) shows the Petri net encoding of the unitary counterpart of $\mathcal{M}$. The arc $(t^1_{v_2}, p^2_{v_2})$ is replaced by the arc $(t^1_{v_2}, p^1_{v_2})$.

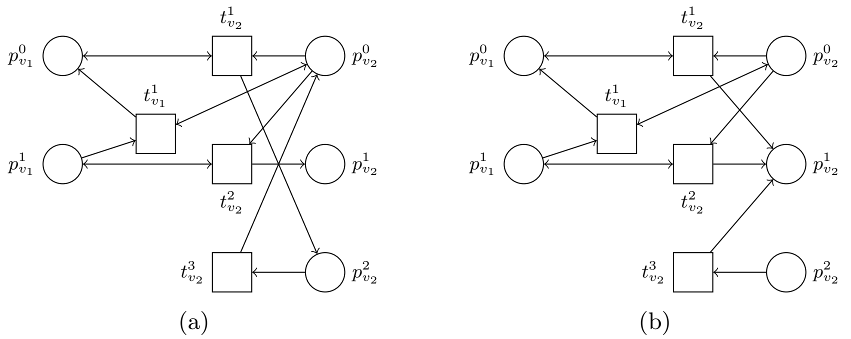

Fig. 3: The Petri net encoding of (a) the general multi-valued network shown in Example 2 and (b) its unitary counterpart.

Encoding of real-world models as 1-safe Petri nets In our work, we consider two primary source formats of multi-valued networks: The SBML-qual format [7] (XML-based) and BMA (BioModelAnalyzer) format [5] (JSON-based). These differ significantly in their structure and capabilities, including the encoding of the multi-valued update functions.

In particular, SBML employs a representation through a list of Boolean terms, where each update function is given in the form:

$$f_i(x) = \begin{cases} y_1 & t^1_{v_i}(x) \\ y_2 & t^2_{v_i}(x) \\ & \dots \\ y_k & \text{else} \end{cases}$$

Here, $y_1, \dots, y_k \in K_i$ and each $t^j_{v_i}$ is a term $\prod_{l=1}^{n} K_l \to \mathbb{B}$ which uses standard equality and inequality propositions on the integer variables. SBML requires that all $t^j_{v_i}$ terms are exclusive, i.e. for any $x \in \mathcal{S}_\mathcal{M}$, it cannot hold that $t^a_{v_i}(x) \wedge t^b_{v_i}(x)$ for any $a \neq b$. This guarantees that the update function is well defined. Note

that it is not required that $y_a \neq y_b$ for $a \neq b$ (i.e. the output values *can* repeat). The outputs also do not need to be exhaustive. However, this is only a technical limitation, as it is easy to transform such f_i into a form where the outputs are all unique and exhaustive. Finally, note that the condition for the "default" value y_k can be easily constructed as $\neg t^1_{v_i}(x) \wedge \ldots \wedge \neg t^{k-1}_{v_i}(x)$.

Meanwhile, the BMA format describes the update functions through a language of algebraic expressions, with support for addition (+), subtraction (−), multiplication (·) and division (/), as well as other special functions like average and rounding. Furthermore, BMA employs a normalisation transformation on function inputs when the input domain differs from the output domain. That is, an input x_j in the range K_j is normalized to the range K_i when used in the update function f_i. As such, while BMA models only admit integer variables, the update functions are more akin to rational functions.

To support both formats, we implement a translation through an intermediate symbolic representation facilitated using binary decision diagrams (BDDs) [6]. In accordance with the Petri net representation, the BDD admits a Boolean variable for every possible level of each variable (places $p^j_{v_i}$ in the PN encoding). Furthermore, every BDD is normalised such that $p^j_{v_i} \Rightarrow \bigwedge_{k \neq j} \neg p^k_{v_i}$. That is, every satisfying valuation of the BDD correctly encodes one integer value of variable v_i. A multi-valued function f_i in then encoded as a list of BDDs $B^0_{f_1}, \ldots, B^{K_i}_{f_i}$, each BDD giving necessary conditions for a particular output level:

$$f_i(x) = \begin{cases} 0 & B^0_{v_i}(x) \\ 1 & B^1_{v_i}(x) \\ & \ldots \\ max(K_i) & B^{K_i}_{v_i}(x) \end{cases}$$

Note that this trivially corresponds to the SBML representation once the model is normalized to include every output exactly once: Every term $t^j_{v_i}$ can be re-encoded as a BDD $B^j_{v_i}$, since integer propositions and logical operators correspond to common operations on BDDs. For the BMA format, we unfortunately have to enumerate the whole function table and re-encode this table into individual BDDs $B^j_{v_i}$.

While there are frameworks which partially support symbolic evaluation of such functions, e.g., algebraic decision diagrams [3], we are not aware of any implementation that would support all the operations required by BMA. As such, we opted for this rather brute-force approach to ensure ideal compatibility with existing BMA toolchain. As we later show in the evaluation, while this introduces additional overhead to model translation, due to the fact that most update functions are of a relatively low arity, the overhead is still manageable.

Using this intermediate representation, we can then construct the Petri net which represents either the general or unitary semantics of the original model. In the general semantics, variable v_i can transition from value a to value b when $p^a_{v_i} \wedge B^b(x)$ holds. We enumerate the satisfying clauses of the resulting decision diagram, each clause giving a partial variable valuation under which the value of

v_i can be updated. Each such clause then results in the creation of one Petri net transition as outlined at the beginning of this section. For the unitary semantics, we implement a transition from a to $a+1$ by enumerating the satisfying clauses of the BDD $p^a_{v_i} \wedge \bigvee_{b>a} B^b_{v_i}(x)$ (symmetrically for transitions into $a-1$).

The resulting Petri net then faithfully encodes the dynamics of the initial multi-valued network. Note that while the number of places in the Petri net is always fixed given a particular set of variables, the number of transitions depends on the structure of the individual update functions and can vary greatly.

2.5 Petri net siphons

Siphons are a static property of Petri nets with many applications in the analysis and control of Petri nets [33]. We recall here the basic definition of a siphon (Definition 4) establishing that to produce something in a siphon you must consume something from the siphon. This definition corresponds to the idea that a siphon is a set of places that once marked remains marked.

Definition 4. *A* siphon *of a Petri net (P, T, W) is a set of places S such that:*

$$\forall t \in T, S \cap succ(t) \neq \emptyset \Rightarrow S \cap pred(t) \neq \emptyset.$$

Note that $\emptyset$ is trivially a siphon.

3 Trap spaces of multi-valued networks

The concept of trap spaces for Boolean networks was pioneered in [29]. It has been proved useful for the Boolean network analysis. It is natural to try to define the similar concept for multi-valued networks, since they are a generalization of Boolean networks. However, this direction has not been explored yet. Hereafter, we shall give a formal definition of trap spaces in multi-valued networks (Subsection 3.1), investigate their properties (Subsection 3.2), and finally discuss their theoretical applications (Subsection 3.3).

3.1 Definition

First, we define the concept of sub-spaces for a multi-valued network.

Definition 5. *A* sub-space *m of a multi-valued network $\mathcal{M} = \langle V, K, F \rangle$ is a mapping m that assigns each node of $\mathcal{M}$ to a non-empty subset of K_i, i.e.,*

$$m(v_i) \subseteq K_i, m(v_i) \neq \emptyset, \forall v_i \in V.$$

A sub-space m is equivalent to the set of states

$$\mathcal{S}_\mathcal{M}[m] := \{s \in \mathcal{S}_\mathcal{M} | \forall v \in V \colon s(v) \in m(v)\}.$$

For example, $m = \{0, 1\}\{0, 2\}$ is a sub-space of the multi-valued network shown in Example 2. m is equivalent a set of four states of the multi-valued network: [0 0], [0 2], [1 0], and [1 2].

Definition 6. *A* sub-space *m of a multi-valued network $\mathcal{M}$ is a* trap space *of $\mathcal{M}$ if it is also a trap set of the state transition graph $(\mathcal{S}_{\mathcal{M}}, \to)$.*

We denote the set of all possible sub-spaces of $\mathcal{M}$ as $\mathcal{S}^{\star}_{\mathcal{M}} = \prod_{i=1}^{n}(\mathbb{P}(K_i) \setminus \emptyset)$ where $\mathbb{P}(K_i)$ denotes the set of all subsets of K_i. Then, we define a partial order $<$ on $\mathcal{S}^{\star}_{\mathcal{M}}$ as: $m < m'$ if and only if $\mathcal{S}_{\mathcal{M}}[m] \subseteq \mathcal{S}_{\mathcal{M}}[m']$ and $\mathcal{S}_{\mathcal{M}}[m] \neq \mathcal{S}_{\mathcal{M}}[m']$. Equivalently, $m < m'$ if and only if $m(v_i) \subseteq m'(v_i), \forall v_i \in V$ and $\exists v_i \in V, m'(v_i) \neq m(v_i)$. From the partial order, we define minimal and maximal trap spaces as follows.

Definition 7. *A trap space m of a multi-valued network $\mathcal{M}$ is* minimal *if and only if there is no trap space $m' \in \mathcal{S}^{\star}_{\mathcal{M}}$ such that $m' < m$.*

Definition 8. *A trap space m of a multi-valued network $\mathcal{M}$ is* maximal *if and only if $S_{\mathcal{M}}[m] \neq S_{\mathcal{M}}$ and there is no trap space $m' \in \mathcal{S}^{\star}_{\mathcal{M}}$ such that $m < m'$ and $S_{\mathcal{M}}[m'] \neq S_{\mathcal{M}}$.*

For illustration, let us consider the general multi-valued network $\mathcal{M}$ shown in Example 2 with the fully asynchronous update scheme. $(\mathcal{S}_{\mathcal{M}}, \hookrightarrow)$ has six trap sets (see Figure 2(a)). We can easily see that $(\mathcal{S}_{\mathcal{M}}, \hookrightarrow)$ has six trap spaces corresponding to the six trap sets as follows:

$$\begin{aligned}
\{[0\ 1]\} &\sim m_1 = \{0\}\{1\},\\
\{[1\ 1]\} &\sim m_2 = \{1\}\{1\},\\
\{[0\ 0], [0\ 2]\} &\sim m_3 = \{0\}\{0, 2\},\\
\{[0\ 1], [1\ 1]\} &\sim m_4 = \{0, 1\}\{1\},\\
\{[0\ 0], [0\ 1], [0\ 2]\} &\sim m_5 = \{0\}\{0, 1, 2\},\\
\{[0\ 0], [0\ 1], [0\ 2], [1\ 0], [1\ 1], [1\ 2]\} &\sim m_6 = \{0, 1\}\{0, 1, 2\}.
\end{aligned}$$

Note that $S_{\mathcal{M}}[m_6] = S_{\mathcal{M}}$. Then, we can see that $(\mathcal{S}_{\mathcal{M}}, \hookrightarrow)$ has three minimal trap spaces (m_1, m_2, and m_3) and two maximal trap spaces (m_4 and m_5). Let $\mathcal{U}$ be the unitary counterpart of $\mathcal{M}$. Similarly, $(\mathcal{S}_{\mathcal{U}}, \hookrightarrow)$ has two minimal trap spaces ($\{0\}\{1\}$ and $\{1\}\{1\}$) and three maximal trap spaces ($\{0, 1\}\{0, 1\}$, $\{0, 1\}\{1, 2\}$, and $\{0\}\{0, 1, 2\}$).

3.2 Properties

Characterization of trap spaces Similar to the characterization of trap spaces in the Boolean case [29], it is possible to characterize trap spaces of a multi-valued network via its update functions. We first denote $\mathrm{dom}(f)$ and $\mathrm{img}(f)$ the domain and the image of the function f, respectively. Let D be a subset of $\mathrm{dom}(f)$. Then $f[D]$ is the restricted function of f under D where $f[D] = \{(x, y) \mid (x, y) \in f, x \in D\}$. We define the image $F[m]$ of a sub-space m under F as a sub-space m' such that $m'(v_i) = \mathrm{img}(f_i[\mathcal{S}_{\mathcal{M}}[m]])$, for every $v_i \in V$.

Theorem 1. *Let $\mathcal{M} = \langle V, K, F \rangle$ be a general multi-valued network. A sub-space m is a trap set of $(\mathcal{S}_{\mathcal{M}}, \to)$ if and only if $F[m] \leq m$.*

Proof. $m \in \mathcal{S}^{\star}_{\mathcal{M}}$ is a trap set of $(\mathcal{S}_{\mathcal{M}}, \rightarrow)$ if and only if there are no $x \in \mathcal{S}_{\mathcal{M}}[m]$ and $y \in \mathcal{S}_{\mathcal{M}} \setminus \mathcal{S}_{\mathcal{M}}[m]$ such that $x \rightarrow y$. This is equivalent to $\mathrm{img}(f_i[\mathcal{S}_{\mathcal{M}}[m]]) \subseteq m(v_i), \forall v_i \in V$, which is equivalent to $F[m] \leq m$ by the definition of the partial order $<$. □

For the case of unitary multi-valued networks, the updated state of a node is not directly obtained from the result of its associated update function on the current state. However, a unitary multi-valued network $\mathcal{U}$ can be equivalent to a general multi-valued network $\mathcal{M}^{\mathcal{U}} = \langle V^{\mathcal{U}}, K^{\mathcal{U}}, F^{\mathcal{U}} \rangle$ where $V^{\mathcal{U}} = V, K^{\mathcal{U}} = K, F^{\mathcal{U}} = \{f^{\mathcal{U}}_1, \dots, f^{\mathcal{U}}_n\}$. Each $f^{\mathcal{U}}_i$ is uniquely obtained from f_i. Formally,

$$f^{\mathcal{U}}_i := \left\{ (x, y') \mid (x, y) \in f_i, y'_i = x_i + \frac{y_i - x_i}{|y_i - x_i|} \right\}.$$

Note that $f^{\mathcal{U}}_i$ is fixed for every $v_i \in V$. In addition, if we want to represent both f_i and $f^{\mathcal{U}}_i$ as expressions, we can consider the use of fuzzy logic [38] or the formulation used in [47].

Theorem 2. *Let $\mathcal{M} = \langle V, K, F \rangle$ be a unitary multi-valued network. A sub-space m is a trap set of $(\mathcal{S}_{\mathcal{M}}, \rightarrow)$ if and only if $F^{\mathcal{U}}[m] \leq m$.*

Proof. This is similar to the proof of Theorem 1 with $F^{\mathcal{U}}$ plays the role of F. □

From Theorems 1 and 2, the trap spaces of a (general or unitary) multi-valued network are independent of the update scheme. This property is similar to the Boolean case as we expected. Hence, trap spaces can be seen as a *static* property of a (general or unitary) multi-valued network.

Separation of minimal trap spaces Hereafter, we prove the separation of minimal trap spaces of a multi-valued network. Specifically, all minimal trap spaces are mutually disjoint. This property is important because we can use it to approximate the set of attractors of the multi-valued network (see Subsection 3.3).

Theorem 3. *Let $\mathcal{M} = \langle V, K, F \rangle$ be a multi-valued network. For any two distinct minimal trap spaces m_1 and m_2 of $\mathcal{M}$, we have that $\mathcal{S}_{\mathcal{M}}[m_1] \cap \mathcal{S}_{\mathcal{M}}[m_2] = \emptyset$.*

Proof. If $\mathcal{M}$ has only one minimal trap space, then the theorem trivially holds. Note that $\mathcal{M}$ always has at least one minimal trap space. Hence, we consider the case that $\mathcal{M}$ has at least two minimal trap spaces.

Consider any two distinct minimal trap spaces m_1 and m_2. Assume that $\mathcal{S}_{\mathcal{M}}[m_1] \cap \mathcal{S}_{\mathcal{M}}[m_2] \neq \emptyset$. Then, there is a sub-space m such that

$$m(v_i) = m_1(v_i) \cap m_2(v_i), m(v_i) \neq \emptyset, \forall v_i \in V.$$

Let s be an arbitrary state in $\mathcal{S}_{\mathcal{M}}[m]$. Clearly, $s \in \mathcal{S}_{\mathcal{M}}[m_1]$ and $s \in \mathcal{S}_{\mathcal{M}}[m_2]$. For any state s' reachable from s regardless of the update scheme of $\mathcal{M}$, we $s' \in \mathcal{S}_{\mathcal{M}}[m_1]$ and $s' \in \mathcal{S}_{\mathcal{M}}[m_2]$ by the definition of a trap set. Then, $s' \in \mathcal{S}_{\mathcal{M}}[m]$, leading to m is trap space of $\mathcal{M}$. Since $m_1 \neq m_2$, $m < m_1$ or $m < m_2$, which contradicts to the minimality of m_1 and m_2. Hence, $\mathcal{S}_{\mathcal{M}}[m_1] \cap \mathcal{S}_{\mathcal{M}}[m_2] = \emptyset$. □

Relations with respect to the Boolean mapping There are several work [15,14] that tries to encode a multi-valued network by a Boolean network. Most of them use the popular Boolean mapping (Van Ham mapping) [26] and focus on unitary (not general) multi-valued networks. In [15], it is proved that the Van Ham Boolean mapping preserves attractors of a unitary multi-valued network with respect to the fully asynchronous update scheme. This Boolean mapping has been implemented in several popular tools in systems biology such as `GINsim` [40] and and `bioLQM` [7]. A natural question is whether the Van Ham Boolean mapping preserves trap spaces of a multi-valued network. Hereafter, we shall show our theoretical findings with respect to trap spaces of multi-valued networks.

First, we show two counterexamples (Examples 3 and 4) with respect to minimal and maximal trap spaces of general multi-valued networks, respectively.

Example 3. Consider the general multi-valued network $\mathcal{M}$ shown in Example 2. Its encoded Boolean network following the Van Ham Boolean mapping is the Boolean network $\mathcal{N}$ shown in Example 1. $\mathcal{M}$ has three minimal trap spaces: $m_1 = \{0\}\{1\}$, $m_2 = \{1\}\{1\}$, and $m_3 = \{0\}\{0, 2\}$. $\mathcal{N}$ has only two minimal trap spaces: $n_1 = 010$ and $n_2 = 110$. n_1 corresponds to m_1 and n_2 corresponds to m_2. However, there is no minimal trap space of $\mathcal{N}$ corresponding to m_3.

Example 4. Consider the general multi-valued network $\mathcal{M}$ shown in Example 2. Its encoded Boolean network following the Van Ham Boolean mapping is the Boolean network $\mathcal{N}$ shown in Example 1. $\mathcal{M}$ has two maximal trap spaces: $m_4 = \{0, 1\}\{1\}$ and $m_5 = \{0\}\{0, 1, 2\}$. $\mathcal{N}$ has three maximal trap spaces: $n_7 = 0\star\star$, $n_8 = \star\star 0$, and $n_9 = \star 1 \star$. n_7 corresponds to m_5. However, n_8 corresponds to the sub-space $\{0, 1\}\{0, 1\}$ and n_9 corresponds to the sub-space $\{0, 1\}\{1, 2\}$. None of them corresponds to m_4.

Second, we show a counterexample (Example 5) with respect to maximal trap spaces of unitary multi-valued networks. For the case of minimal trap spaces, we show that the Van Ham Boolean mapping preserves all the minimal trap spaces of a unitary multi-valued network. For the details, see Appendix A.

Example 5. Consider the unitary multi-valued network $\mathcal{U}$ where $V = \{v_1, v_2\}$, $K_1 = K_2 = \{0, 1, 2\}$, and

$$F = \begin{cases} f_1 = \begin{cases} 0 \text{ if } v_1 = 0 \text{ or } v_2 = 0, \\ 1 \text{ if } v_1 = 1 \text{ and } v_2 = 1 \text{ or } v_1 = 1 \text{ and } v_2 = 2 \text{ or } v_1 = 2 \text{ and } v_2 = 1, \\ 2 \text{ otherwise.} \end{cases} \\ f_2 = \begin{cases} 0 \text{ if } v_1 = 2 \text{ or } v_2 = 2, \\ 1 \text{ if } v_1 = 1 \text{ and } v_2 = 0 \text{ or } v_1 = 1 \text{ and } v_2 = 1 \text{ or } v_1 = 0 \text{ and } v_2 = 1, \\ 2 \text{ otherwise.} \end{cases} \end{cases}$$

Let $\mathcal{N}$ be the encoded Boolean network of $\mathcal{U}$ by the Van Ham Boolean mapping. $\mathcal{U}$ has two maximal trap spaces: $u_1 = \{0, 1\}\{0, 1, 2\}$ and $u_2 = \{0, 1, 2\}\{0, 1\}$. $\mathcal{N}$ has three maximal trap spaces: $n_1 = \star 0 \star\star$, $n_2 = \star\star\star 0$, and $n_3 = 0 \star\star\star$. n_1

and n_2 correspond to u_1 and u_2, respectively. In $\mathcal{U}$, $\{0\}\{0,1,2\}$ is a trap space smaller than the trap space u_1, thus it is not maximal. n_3 corresponds to this trap space. However, in $\mathcal{N}$, the presence of an inadmissible state 01 for node v_1 makes n_3 maximal.

Finally, we prove that the Van Ham Boolean mapping preserves fixed points of both general and unitary multi-valued networks. It is worth recalling that a fixed point of a multi-valued network is a special trap space. Specifically, a trap space m is a fixed point if and only if $|m(v_i)| = 1$ for every node $v_i \in V$.

Theorem 4. *Let $\mathcal{M} = \langle V, K, F\rangle$ be a (general or unitary) multi-valued network and $\mathcal{N} = \langle V^{\mathcal{N}}, F^{\mathcal{N}}\rangle$ be its encoded Boolean network using the Van Ham Boolean mapping. Then there is a bijection between the set of fixed points of $\mathcal{M}$ and that of $\mathcal{N}$.*

Proof. For the case of unitary multi-valued networks, this theorem is a direct consequence of the theoretical result showing that the set of attractors of $\mathcal{M}$ is equivalent to that of $\mathcal{N}$ with respect to the fully asynchronous update scheme [15]. The reason is that fixed points of a unitary multi-valued network are independent of the update scheme. Now, we only consider general multi-valued networks. Let $\mathcal{U}$ be the unitary counterpart of $\mathcal{M}$. Following the update semantics of general and unitary multi-valued networks, a fixed point of $\mathcal{M}$ is also a fixed point of $\mathcal{U}$ and vice versa. Hence, there is also a bijection between the set of fixed points of $\mathcal{M}$ and that of $\mathcal{N}$. □

3.3 Theoretical applications

Model reduction Let m be a trap space of a multi-valued network $\mathcal{M} = \langle V, K, F\rangle$. We define a new multi-valued network $\mathcal{M}^m = \langle V^m, K^m, F^m\rangle$ such that

$$\begin{aligned} V^m &= V \setminus \{v_i \mid v_i \in V, |m(v_i)| = 1\}, \\ K^m &= \{K_i^m \mid v_i^m \in V^m, K_i^m = m(v_i^m)\}, \\ F^m &= \{f_i^m \mid v_i^m \in V^m, f_i^m = f_i[\mathcal{S}_{\mathcal{M}}[m]]|_A\}, \end{aligned}$$

where $A = \prod_{j=1}^{|V^m|} K_{i_j}^m$ and $f_i[\mathcal{S}_{\mathcal{M}}[m]]|_A$ is the restriction of $f_i[\mathcal{S}_{\mathcal{M}}[m]]$ to A. We can easily see that $\mathcal{M}^m$ captures the whole dynamics of the part of state space of $\mathcal{M}$ contained in m. Specifically, a transition of any two states in $\mathcal{S}_{\mathcal{M}}[m]$ is equivalent to a transition in the state transition graph of $\mathcal{M}^m$. This property makes a natural model reduction technique.

Attractor approximation A trap space is a trap set and an attractor is a minimal trap set of a multi-valued network $\mathcal{M}$. Therefore, a trap space contains at least one attractor of $\mathcal{M}$ regardless of the update scheme. However, two distinct trap spaces can overlap. We have proved in Subsection 3.2 that two any minimal trap spaces are disjoint. Hence, a minimal trap space contains at least

one attractor and two any attractors contained in two distinct minimal trap spaces are disjoint.

With the above property, the set of minimal trap spaces of $\mathcal{M}$ can be an approximation of the set of attractors of $\mathcal{M}$ regardless of the update scheme. See Examples 6, 7, and 8 for three cases where the set of minimal trap spaces is not exactly the set of attractors. Note that a minimal trap space m cannot contain both fixed points and cyclic attractors. Indeed, if it holds, then there is another trap space smaller than m because a fixed point is a special trap space, which contradicts to the minimality of m. Furthermore, the set of attractors of $\mathcal{M}$ contained in m is equivalent to the set of attractors of the reduced multi-valued network obtained by the above model reduction. This can be helpful for attractor detection in multi-valued networks.

Example 6. Consider the general multi-valued network $\mathcal{M} = \langle V, K, F \rangle$ where:

- $V = \{v_1, v_2\}$
- $K_1 = \{0, 1\}$, $K_2 = \{0, 1, 2\}$
-

$$F = \begin{cases} f_1 = \begin{cases} 0 \text{ if } v_1 = 0 \text{ and } v_2 = 1 \text{ or } v_1 = 1 \text{ and } v_2 = 0 \text{ or } v_1 = 1 \text{ and } v_2 = 2, \\ 1 \text{ otherwise.} \end{cases} \\ f_2 = \begin{cases} 0 \text{ if } v_1 = 0 \text{ or } v_2 = 1 \text{ or } v_1 = 1 \text{ and } v_2 = 0, \\ 1 \text{ if } v_1 = 0 \text{ or } v_2 = 0 \text{ or } v_1 = 1 \text{ and } v_2 = 2, \\ 2 \text{ otherwise.} \end{cases} \end{cases}$$

Figure 4(a) shows the state transition graph of $\mathcal{M}$ under the fully asynchronous update scheme. $\mathcal{M}$ has only one minimal trap space that is {0, 1}{0, 1, 2}. This minimal trap space contains two asynchronous attractors of $\mathcal{M}$: {[0 0], [1 0], [0 1]} and {[1 2], [1 1], [0 2]}.

Example 7. Consider the general multi-valued network $\mathcal{M} = \langle V, K, F \rangle$ where:

- $V = \{v_1, v_2\}$
- $K_1 = \{0, 1\}$, $K_2 = \{0, 1, 2\}$
-

$$F = \begin{cases} f_1 = \begin{cases} 0 \text{ if } v_1 = 0 \text{ and } v_2 = 1 \text{ or } v_1 = 1 \text{ and } v_2 = 0 \text{ or } v_1 = 1 \text{ and } v_2 = 1, \\ 1 \text{ otherwise.} \end{cases} \\ f_2 = \begin{cases} 0 \text{ if } v_1 = 0 \text{ or } v_2 = 1 \text{ or } v_1 = 1 \text{ and } v_2 = 0, \\ 1 \text{ if } v_1 = 0 \text{ or } v_2 = 0 \text{ or } v_1 = 1 \text{ and } v_2 = 1, \\ 2 \text{ otherwise.} \end{cases} \end{cases}$$

Figure 4(b) shows the state transition graph of $\mathcal{M}$ under the fully asynchronous update scheme. $\mathcal{M}$ has only one minimal trap space that is {0, 1}{0, 1, 2}. This minimal trap space contains the sole asynchronous attractor of $\mathcal{M}$: {[0 0], [1 0], [0 1]}. [1 1], [1 2], [0 2] are three states included in the minimal trap space, but they do not belong to the asynchronous attractor.

Example 8. Consider the general multi-valued network $\mathcal{M} = \langle V, K, F\rangle$ where:

- $V = \{v_1, v_2\}$
- $K_1 = \{0, 1\}$, $K_2 = \{0, 1, 2\}$
-

$$F = \begin{cases} f_1 = \begin{cases} 0 \text{ if } v_1 = 0 \text{ and } v_2 = 1 \text{ or } v_1 = 1 \text{ and } v_2 = 0 \\ 1 \text{ otherwise.} \end{cases} \\ f_2 = \begin{cases} 0 \text{ if } v_1 = 0 \text{ or } v_2 = 1 \text{ or } v_1 = 1 \text{ and } v_2 = 0, \\ 1 \text{ if } v_1 = 0 \text{ or } v_2 = 0 \text{ or } v_1 = 1 \text{ and } v_2 = 2, \\ 2 \text{ otherwise.} \end{cases} \end{cases}$$

Figure 4(c) shows the state transition graph of $\mathcal{M}$ under the fully asynchronous update scheme. $\mathcal{M}$ has only one minimal trap space that is {1}{1, 2}. This minimal trap space contains an asynchronous attractor of $\mathcal{M}$: {[1 1], [1 2]}. However, $\mathcal{M}$ has another asynchronous attractor (i.e., {[0 0], [1 0], [0 1]}) that is not included in any minimal trap space of $\mathcal{M}$.

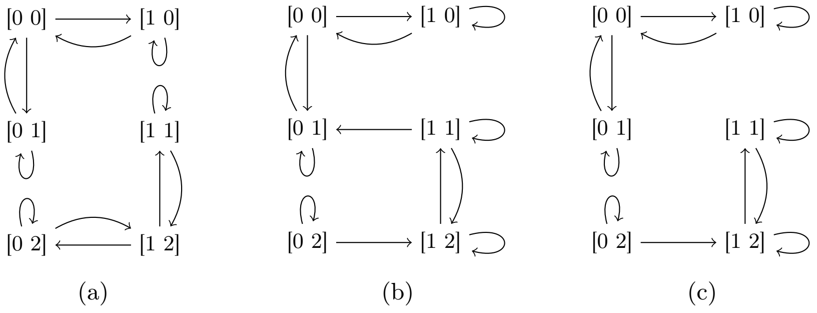

Fig. 4: State transition graphs under the fully asynchronous update scheme of (a) the general multi-valued network shown in Example 6, (b) the general multi-valued network shown in Example 7, and (c) the general multi-valued network shown in Example 8, respectively.

Control and decision making Control of biological systems is one of the central issues in systems biology with various applications in systems-based drug discovery and cancer treatment [28,2]. Roughly speaking, this problem can be defined as the design of intervention strategies (control policies) to beneficially alter the dynamics of the considered system [2]. For example, in the Boolean network model of a gene regulatory network, we can control one or more genes (e.g., knockout or overexpression) such that the model moves out of undesirable states (e.g., disease or cancerous states) and moves into desirable ones (e.g., healthy or normal states). Such types of states are associated to attractors as a particular attractor (or set of attractors and their associated basins) supports states

that resemble a pathological phenotype. Since biological systems are often highly non-linear, existing methods in control theory cannot be directly applied to this control problem. Hence, it has attracted much attention from many research communities. Note that there are many formulations for the control problem in biological systems with a variety of approaches and goals. We refer the reader to [19,46] and references therein for more detailed discussions.

Regarding the control problem of Boolean network models of biological systems, there are several methods [19,12] using trap spaces of a Boolean network to compute control strategies that guide this Boolean network converge into a desirable phenotype (e.g., a minimal trap space). These methods are independent of the update scheme and have been shown useful for control of biological systems [12]. Since we have defined the concept of trap spaces for multi-valued networks, it is potentially possible to extend these methods to those for multi-valued networks. In addition, the work [45] uses the concept of stable motifs to build the succession diagram of a Boolean network that serves as a summary of the decisions in the network dynamics that lead to successively more restrictive nested stable motifs. The succession diagram is useful for control and decision making of this Boolean network. It has been shown that a stable motif of a Boolean network is equivalent to a maximal trap space of this Boolean network. Analogously, a stable motif of a multi-valued network [20] is equivalent to a maximal trap space of this multi-valued network. Hence, it is potentially possible to apply this stable motifs-based approach to multi-valued networks.

Extending the existing trap spaces-based methods for control of Boolean networks to those for control of multi-valued networks are potential and promising. However, it is out of scope of the present article. We leave this direction as future work.

4 Trap spaces as conflict-free siphons

Recently, we have explored a connection between trap spaces of a Boolean network and siphons of its Petri net encoding [55]. Once we have formally defined the concept of trap spaces for multi-valued networks in Subsection 3.1, we shall generalize this connection to that for trap spaces of multi-valued networks and siphons of Petri nets (Subsection 4.1). Not only a natural generalization, we in addition add more substantially computational and theoretical results because of the different characteristics of multi-valued networks as compared to Boolean networks.

4.1 Relations

First, we add a definition related to any set of places of a Petri net encoding a multi-valued network, and notably a siphon of such a net.

Definition 9. *A set of places of Petri net $\mathcal{P}$ encoding multi-valued network $\mathcal{M} = \langle V, K, F \rangle$ is* conflict-free *if it does not contain all the places corresponding*

the possible states of the same node *of $\mathcal{M}$. Then, a conflict-free siphon S is said to be* maximal *if and only if there is no conflict-free siphon S' such that $S \subset S'$. A conflict-free siphon S is said to be* minimal *if and only if $S \neq \emptyset$ and there is no conflict-free siphon S' such that $S' \subset S$ and $S' \neq \emptyset$.*

Then, we define the concept of *mirror* as follows.

Definition 10. *Let m be a sub-space of multi-valued network $\mathcal{M} = \langle V, K, F \rangle$. A* mirror *of m is a set of places S in the Petri net encoding $\mathcal{P}$ of $\mathcal{M}$ such that:*

$$\forall v \in V, \forall i \in K_v, i \in m(v) \Leftrightarrow p_v^i \notin S \text{ and } i \in K_v \setminus m(v) \Leftrightarrow p_v^i \in S.$$

Intuitively, every conflict-free siphon of $\mathcal{P}$ represents a mirror of some sub-space m of $\mathcal{M}$. Once the places in a siphon are unmarked, they remain unmarked. This precisely corresponds to the closed property of a trap space (see Subsection 3.1). Hence, a conflict-free siphon of $\mathcal{P}$ corresponds to a trap space of $\mathcal{M}$. In addition, the maximality (resp. minimality) of a conflict-free siphon is equivalent to as many (resp. less) fixed values as possible, hence the minimality (resp. maximality) of a trap space. Hereafter, we formally prove these relations.

Theorem 5. *Let $\mathcal{M} = \langle V, K, F \rangle$ be a multi-valued network and $\mathcal{P}$ be its Petri net encoding. A sub-space m is a trap space of $\mathcal{M}$ if and only if its mirror S is a conflict-free siphon of $\mathcal{P}$.*

Proof. First, we show that if m is a trap space of $\mathcal{M}$, then S is a conflict-free siphon of $\mathcal{P}$ (*). If $m(v_i) = K_i, \forall v_i \in V$, then $S = \emptyset$ is trivially a conflict-free siphon of $\mathcal{P}$. Thus, we consider the case that $S \neq \emptyset$. Assume that S is not a siphon of $\mathcal{P}$. Then, there is a transition $t \in T$ such that $S \cap succ(t) \neq \emptyset$ but $S \cap pred(t) = \emptyset$. This implies that there is a place $p_{v_i}^{j_1} \in S$ such that $p_{v_i}^{j_1} \in succ(t)$ but $p_{v_i}^{j_1} \notin pred(t)$. By the characteristics of the encoding [10], there is a directional arc from t to $p_{v_i}^{j_1}$ and a directional arc from $p_{v_i}^{j_2}$ to t with $j_2 \neq j_1$. Then $p_{v_i}^{j_2} \notin S$ because $S \cap pred(t) = \emptyset$. We follow the following procedure to find a state $s \in \mathcal{S}_{\mathcal{M}}[m]$ such that $M_s(p) = 1, \forall p \in pred(t)$ where M_s is the corresponding marking in $\mathcal{P}$ of s. It is worth recalling that $pred(t)$ cannot contain two places corresponding to the same node of $\mathcal{M}$. For every place $p_{v_l}^k \in pred(t)$ (implying $p_{v_l}^k \notin S$), we set $s(v_l) = k$. Then $M_s(p_{v_l}^k) = 1$ by the characteristics of the encoding [10]. By the definition of a mirror, $k \in m(v_l)$ because $p_{v_l}^k \notin S$. For the remaining nodes v of $\mathcal{M}$, we set $s(v)$ to any value in $m(v)$. Overall, there is always such a state s. Then t is enabled at marking M_s. Its firing leads to a new marking M_s' such that $M_s'(p_{v_i}^{j_1}) = 1$. Let s' be the corresponding state in $\mathcal{M}$ of M_s'. By the characteristics of the encoding [10], $s'(v_i) = j_1$. We have $j_1 \notin m(v_i)$ because $p_{v_i}^{j_1} \in S$. Therefore, $s' \notin \mathcal{S}_{\mathcal{M}}[m]$. For any firing scheme of $\mathcal{P}$, the firing of t always happens. Since a firing scheme of $\mathcal{P}$ is equivalent to an update scheme of $\mathcal{M}$, s can escape from the trap space m for any update scheme of $\mathcal{M}$, which contradicts to the property of a trap space. Hence, S is a siphon of $\mathcal{P}$. By the definition of a mirror, S is also a conflict-free one.

Second, we show that if S is a conflict-free siphon of $\mathcal{P}$, then m is a trap space of $\mathcal{M}$ (**). By the definition of a mirror, m is a sub-space of $\mathcal{M}$. Let s be an

arbitrary state in $\mathcal{S}_{\mathcal{M}}[m]$ and M_s be its corresponding marking in $\mathcal{P}$. If there is a place $p_{v_i}^j \in S$ such that $M_s(p_{v_i}^j) = 1$, then $s(v_i) = j$. Since $s(v_i) \in m(v_i)$, $p_{v_i}^j \notin S$ by the definition of a mirror, which is a contradiction. Hence, $M_s(p) = 0, \forall p \in S$. In any marking M'_s reachable from M_s regardless of the firing scheme of $\mathcal{P}$, we have $M'_s(p) = 0, \forall p \in S$ by the dynamical property on markings of a siphon [33]. Let s' be the corresponding state in $\mathcal{M}$ of M'_s. If $s'(v_i) = k \notin m(v_i)$, then $p_{v_i}^k \in S$ by the definition of a mirror and $M'_s(p_{v_i}^k) = 1$ by the characteristics of the encoding [10], which is a contradiction. Hence, $s'(v_i) \in m(v_i), \forall v_i \in V$. Then, $s' \in \mathcal{S}_{\mathcal{M}}[m]$. By the definition of a trap space and the arbitrariness of s, m is a trap space of $\mathcal{M}$.

From (*) and (**), we can conclude the proof. □

From the proof of Theorem 5, we can see that this theorem still holds for any update scheme of the multi-valued network. The Petri net encoding of a multi-valued network is independent of its update scheme and siphons are a static property of a Petri net. Hence, trap spaces of a multi-valued network are independent of its update scheme. Theorem 5 provides us another way complementary to the proofs of Theorems 1 and 2 for proving this property of trap spaces in multi-valued networks. This exhibits the very first theoretical application of the connection between trap spaces of multi-valued networks and siphons of Petri nets.

Theorem 6. *Let $\mathcal{M} = \langle V, K, F \rangle$ be a multi-valued network and $\mathcal{P}$ be its Petri net encoding. A sub-space m is a minimal trap space of $\mathcal{M}$ if and only if its mirror S is a maximal conflict-free siphon of $\mathcal{P}$.*

Proof. First, we show that if m is a minimal trap space of $\mathcal{M}$, then S is a maximal conflict-free siphon of $\mathcal{P}$ (*). Since m is a trap space of $\mathcal{M}$, S is a conflict-free siphon of $\mathcal{P}$ by Theorem 5. Assume that S is not maximal. Then there is another conflict-free siphon S' such that $S \subset S'$. By Theorem 5, there is a trap space m' corresponding to S'. Following the definition of a mirror, $m'(v) \subseteq m(v), \forall v \in V$, thus $m' < m$. This is a contradiction because m is a minimal trap space. Hence, S is a maximal conflict-free siphon of $\mathcal{P}$.

Second, we show that if S is a maximal conflict-free siphon of $\mathcal{P}$, then m is a minimal trap space of $\mathcal{M}$ (**). Since S is a conflict-free siphon of $\mathcal{P}$, m is a trap space of $\mathcal{M}$ by Theorem 5. Assume that m is not minimal. Then there is another trap space m' such that $m' < m$. By the definition of the partial order $<$, $m'(v) \subseteq m(v), \forall v \in V$ and there is a $v \in V$ such that $m'(v) \subset m(v)$. Let S' be the mirror of m'. S' is a conflict-free siphon by Theorem 5. Following the definition of a mirror, $S \subset S'$, which is a contradiction because S is a maximal conflict-free siphon. Hence, m is a minimal trap space of $\mathcal{M}$.

From (*) and (**), we can conclude the proof. □

Theorem 7. *Let $\mathcal{M} = \langle V, K, F \rangle$ be a multi-valued network and $\mathcal{P}$ be its Petri net encoding. A sub-space m is a maximal trap space of $\mathcal{M}$ if and only if its mirror S is a minimal conflict-free siphon of $\mathcal{P}$.*

Proof. Let $\mathcal{S}^\star_{\mathcal{M}}$ be the set of all possible sub-spaces of $\mathcal{M}$ and $\mathcal{S}^\star_{\mathcal{P}}$ be the set of all possible conflict-free siphons of $\mathcal{P}$. Let ε be the special sub-space where $\varepsilon(v_i) = K_i, \forall v_i \in V$. By the definition of a mirror, the mirror of ε is an empty set. Note that $\emptyset \in \mathcal{S}^\star_{\mathcal{P}}$. By considering only $\mathcal{S}^\star_{\mathcal{M}} \setminus \{\varepsilon\}$ and accordingly $\mathcal{S}^\star_{\mathcal{P}} \setminus \{\emptyset\}$, we can conclude the proof by using the similar reasoning shown in the proof of Theorem 6.

For illustration, let us consider the general multi-valued network $\mathcal{M}$ shown in Example 2. Its Petri net encoding $\mathcal{P}_{\mathcal{M}}$ is shown in Figure 3(a). $\{p^0_{v_2}, p^1_{v_2}, p^2_{v_2}\}$ is a siphon of $\mathcal{P}_{\mathcal{M}}$, however it is not a conflict-free one. $\mathcal{P}_{\mathcal{M}}$ has all six conflict-free siphons corresponding to the six trap spaces of $\mathcal{M}$ as follows:

$$\begin{aligned}
S_1 = \{p^1_{v_1}, p^0_{v_2}, p^2_{v_2}\} &\sim m_1 = \{0\}\{1\},\\
S_2 = \{p^0_{v_1}, p^0_{v_2}, p^2_{v_2}\} &\sim m_2 = \{1\}\{1\},\\
S_3 = \{p^1_{v_1}, p^1_{v_2}\} &\sim m_3 = \{0\}\{0,2\},\\
S_4 = \{p^0_{v_2}, p^2_{v_2}\} &\sim m_4 = \{0,1\}\{1\},\\
S_5 = \{p^1_{v_1}\} &\sim m_5 = \{0\}\{0,1,2\},\\
S_6 = \emptyset &\sim m_6 = \{0,1\}\{0,1,2\}.
\end{aligned}$$

$\mathcal{P}_{\mathcal{M}}$ has three maximal conflict-free siphons (S_1, S_2, and S_3) and two minimal conflict-free siphons (S_4 and S_5). We can easily see the one-to-one correspondence between the set of maximal (resp. minimal) conflict-free siphons and the set of minimal (resp. maximal) trap spaces. Let $\mathcal{U}$ be the unitary counterpart of $\mathcal{M}$. Its Petri net encoding $\mathcal{P}_{\mathcal{U}}$ is shown in Figure 3(b). $\{p^1_{v_1}, p^1_{v_2}\}$ is not a siphon of $\mathcal{P}_{\mathcal{U}}$ because transition $t^1_{v_2}$ puts tokens into this set but it does not remove tokens from this set. This is the reason why $\{0\}\{0,2\}$ is not a trap space of $\mathcal{U}$.

4.2 Trap space computation

Hereafter, we propose several methods for computing several types of trap spaces in multi-valued networks. These proposed methods mainly rely on the theoretical results presented in Section 3.

Minimal and maximal trap space computation Besides minimal trap spaces, maximal trap spaces also play a crucial role in the analysis and control of biological systems modeled by Boolean networks [29,46]. Notably, the concept of stable motifs is the primary key for various excellent work in logical modeling from Reka Albert's group [62,20,46]. More specifically, they use stable motifs, which are equivalent to maximal trap spaces [46], to build the succession diagram of a Boolean network, which is a hierarchy of stable motifs and encompasses the entire repertoire of decisions that the model is capable of making [62]. Once the succession diagram is successfully built, it can be used to not only identify attractors of this Boolean network under the fully asynchronous update scheme [46] but also to control the dynamics of this Boolean network regardless

of its update scheme [62,45]. In addition, the succession diagram's leaf nodes are the minimal trap spaces of the Boolean network [46]. Hence, we can see that maximal trap spaces have broader applicability than minimal trap spaces. In particular, using the stable motifs-based methods, many studies [50,61,16,36] have obtained important biological results, notably, the suppression of epithelial-to-mesenchymal transition [50], hair follicles cell fate control [16]. Recall that a stable motif of a multi-valued network [20] is equivalent to a maximal trap space of this multi-valued network. Analogously, maximal trap spaces also play a crucial the analysis and control of biological systems modeled by multi-valued networks. Hence, it is of great importance to compute maximal trap spaces in multi-valued networks.

By Theorem 6 (resp. Theorem 7), we can reduce the problem of computing all minimal (resp. maximal) trap spaces of a multi-valued network to the problem of computing all maximal (resp. minimal) conflict-free siphons of its Petri net encoding. It is noted that there are no existing methods specifically designed for computing maximal conflict-free siphons (even maximal siphons) of a Petri net. There are some existing methods [39] for computing minimal siphons of a Petri net. Although they do not directly support the minimal conflict-free siphon computation, we can adjust them a little to compute minimal conflict-free siphons. For example, with the SAT-based method [39], we can add more constraints to the SAT formula to express the conflict-freeness. Since we focus on both minimal and maximal trap spaces of multi-valued networks, we here propose a unified method based on Answer Set Programming (ASP) [23] for computing both maximal and minimal conflict-free siphons of a Petri net. It is worth noting that ASP has been proved very efficient for computing trap spaces (especially the case of many solutions) of Boolean networks [29,42,55]. The details of the proposed method (named `PN-TS`) shall be given as follows.

First, we show the characterization of all generic siphons of the encoded Petri net $\mathcal{P} = (P, T, W)$. This characterization is the same as that shown in Section 4 of [55]. We recall it here for making the present article self-contained. Suppose that S is a generic siphon of $\mathcal{P}$. If a place p should belong to S, then by definition all the transitions in $pred(p)$ must belong to $succ(S)$. Note that $succ(S) = \bigcup_{p \in S} succ(p)$. A transition t belongs to $succ(S)$ if and only if there is at least one place p' in S such that $p' \in pred(t)$. Hence, for each transition $t \in pred(p)$, we can state that

$$p \in S \Rightarrow \bigvee_{p' \in pred(t)} p' \in S. \tag{1}$$

By definition, the final system fully characterizes all generic siphons of the encoded Petri net.

Second, to make S to be a conflict-free siphon, we need to add to the system the rule

$$\bigwedge_{j \in K_i} (p^j_{v_i} \in S) = 0 \tag{2}$$

for every node $v_i \in V$. This rule represents that siphon S cannot contain all the places corresponding to all the possible values of the same node.

Then, we translate the above characterization into the ASP $\mathcal{L}$ as follows. The set $\mathcal{A}$ of all atoms in $\mathcal{L}$ is the set of places of $\mathcal{P}$, i.e., $\mathcal{A} = \{p_{v_i}^j \mid v_i \in V, j \in K_i\}$. For each pair (p, t) where $p \in P, t \in T, t \in \mathit{pred}(p)$, we translate the rule (1) into the ASP rule

$$a_1; \dots; a_k \,\text{:-}\, a.$$

where $a \in \mathcal{A}$ is the atom representing place p and $\{a_1, \dots, a_k\} \subseteq \mathcal{A}$ is the set of atoms representing places in $\mathit{pred}(t)$. The rule (2) is translated into the ASP rule

$$\text{:-}\, p_{v_i}^{k_1}, \dots, p_{v_i}^{k_{|K_i|}}.$$

for each $v_i \in V$ where $\{k_1, \dots, k_{|K_i|}\} = K_i$. This ASP rule guarantees that all the places representing the same node in $\mathcal{M}$ never belong to the same siphon of $\mathcal{P}$, representing the conflict-freeness. Naturally, a Herbrand model (see, e.g, [23]) of $\mathcal{L}$ is equivalent to a conflict-free siphon of $\mathcal{P}$. To guarantee that a Herbrand model is also a stable model (an answer set), we need to add to $\mathcal{L}$ the $|K_i|$ choice rules

$$\{p_{v_i}^j\}.$$

with $j \in K_i$ for each $v_i \in V$.

Now, a solution (simply an answer set) $A \subseteq \mathcal{A}$ of $\mathcal{L}$ is equivalent to a conflict-free siphon S of $\mathcal{P}$, thus a trap space m of $\mathcal{M}$. The conversion from A to m is straightforward following the mirror function in Definition 10. Formally,

$$m(v_i) = \{j \mid j \in K_i, p_{v_i}^j \notin A\}$$

for every node $v_i \in V$. Computing multiple answer sets is built into ASP solvers and the solving collection POTASSCO [23] also features the option to find set-inclusion maximal/minimal answer sets with respect to the set of atoms. Naturally, a set-inclusion maximal answer set of $\mathcal{L}$ is equivalent to a maximal conflict-free siphon of $\mathcal{P}$, thus a minimal trap space of $\mathcal{M}$. By using this built-in option, we can compute all the set-inclusion maximal answer sets of $\mathcal{L}$ (resp. all the minimal trap spaces of $\mathcal{M}$) in one execution. For the case of minimal conflict-free siphons, we need to exclude the special answer set $\emptyset$ (corresponding to the empty siphon and the special trap space ε) from the solution space. To do this, we add to the encoded ASP the following rule

$$p_{v_1}^{k_1^1}; \dots; p_{v_1}^{k_{|K_1|}^1}; \dots; p_{v_n}^{k_1^n}; \dots; p_{v_n}^{k_{|K_n|}^n}.$$

where $\{k_1^i; \dots; k_{|K_i|}^i\} = K_i$ for every $v_i \in \{v_1, \dots, v_n\}$. By using the built-in option of ASP solvers with respect to set-inclusion minimal answer sets, we can compute all the set-inclusion minimal answer sets of $\mathcal{L}$ (resp. all the maximal trap spaces of $\mathcal{M}$) in one execution. It is worth noting that the above encoding does not requires the assumption on the domain sets (i.e., $K_i = \{0, \dots, |K_i|-1\}$), which shows its broader applicability.

Fixed point computation A fixed point of a multi-valued network is a special trap space. Historically, fixed point computation is the fundamental and the most popular analysis for biological systems modeled by multi-valued networks [22,47,52,1]. However, to date, such analysis remains a very useful tool in understanding the behavior of complex biological models. On the one hand, the full computation of complex attractors remains intractable in some cases, especially for large-scale models [49]. On the other hand, for many biological systems, the expected long-term behavior is not cyclic such as the cell cycle and circadian rhythms, but rather a stabilization to an observable phenotype (e.g., cell differentiation, apoptosis, proliferation, signal transduction, protein transcription). We can easily find several recent studies (see, e.g., [18,13,57]) using fixed points as main validation. Furthermore, the fixed point computation plays the crucial role in the several state-of-the-art methods for computing complex attractors of Boolean networks under the fully asynchronous update scheme [25,56], which can be used to compute attractors of multi-valued networks because the Van Ham Boolean mapping preserves attractors of unitary multi-valued networks under the fully asynchronous update scheme [15]. Hence, we here propose two possible ways to compute all fixed points of a multi-valued network. Both rely on the Petri net encoding of this multi-valued network.

The first one called `PN-F-1` relies on the characterization of trap spaces of $\mathcal{M}$. Indeed, a fixed point s is also a trap space where $|s(v_i)| = 1$ for every $v_i \in V$. To guarantee this constraint in the encoded ASP $\mathcal{L}$ that characterizes all trap spaces of $\mathcal{M}$, we add to $\mathcal{L}$ the following rule

$$k\{p_{v_i}^{k_1}; \ldots ; p_{v_i}^{k_{|K_i|}}\}.$$

for every $v_i \in V$ where $k = |K_i| - 1$ and $\{k_1, \ldots, k_{|K_i|}\} = K_i$. This rule combining with the ASP rule expressing the conflict-freeness ensures that for any answer set and for every node $v_i \in V$, there is exactly one atom corresponding to v_i and not belonging to the answer set. Now, the set of all answer sets of $\mathcal{L}$ is equivalent to the set of all fixed points of $\mathcal{M}$. The conversion is similar to that for the case of trap spaces.

The second one called `PN-F-2` relies on the characterization of deadlocks of the Petri net encoding of $\mathcal{M}$. Because of the characteristics of the encoding [10], a fixed point of $\mathcal{M}$ is equivalent to a deadlock of its Petri net encoding $\mathcal{P}$. Hence, we here construct a new ASP $\mathcal{L}_{\mathcal{P}}$ that characterizes all deadlocks of $\mathcal{P}$. First, the set of atoms of $\mathcal{L}_{\mathcal{P}}$ is the same as that of $\mathcal{L}$. Second, a marking M of $\mathcal{P}$ is a deadlock if and only if there is no enabled transition at M. Formally, the condition

$$\forall t \in T, pred(t) \nsubseteq M$$

holds. Since $pred(t) \nsubseteq M$ is equivalent to there is that the condition $\forall p \in pred(t), M(p) > 0$ cannot hold, we add to $\mathcal{L}_{\mathcal{P}}$ the ASP rule

$$:\!-\, a_1; \ldots ; a_k.$$

for every $t \in T$ where $\{a_1, \ldots, a_k\}$ is the set of atoms corresponding to the set of places $pred(t)$. Finally, we add to $\mathcal{L}_\mathcal{P}$ the ASP rule

$$1\{p_{v_i}^{k_1}; \ldots; p_{v_i}^{k_{|K_i|}}\}1.$$

for every $v_i \in V$ where $\{k_1, \ldots, k_{|K_i|}\} = K_i$. This rule expresses the constraint that every marking of $\mathcal{P}$ must satisfy that only one place corresponding to v_i is marked at the marking [10]. Now, an answer set $A \subseteq \mathcal{A}$ of $\mathcal{L}_\mathcal{P}$ is equivalent to a deadlock M of $\mathcal{P}$, thus a fixed point s of $\mathcal{M}$. The conversion from A to s is opposite to the conversion from an answer set of $\mathcal{L}$ to a fixed point of $\mathcal{M}$ (see the method `PN-F-1`). Specifically,

$$s(v_i) = j, p_{v_i}^j \in A$$

for every node $v_i \in V$.

5 Case study

To demonstrate the usefulness of `trap-mvn`, we look at existing methodologies centred around attractors and how such methodologies can be enriched by added focus on trap spaces. In particular, we look at the findings of [32], where a study of a large computational model was performed using the tool `BMA` [5] in order to reveal novel therapeutic targets for breast cancer.

5.1 Heterogeneity of Myc expression in breast cancer

The Myc transcription factor is one of the key coordinators in cell proliferation and regeneration [31]. As such, oncogenic deregulations of Myc are commonplace in many cancers. Particularly in breast cancer, Myc is typically one of the most overexpressed genes [59].

Still, most tumours have been shown to consist of several genetically distinct mutants, only some of which exhibit Myc overexpressions [24,27]. This heterogeneity can impede possible treatments, e.g., those that only target a particular subclass of all mutants. However, it can also enable new treatments that target the interplay or cooperation between the various mutants [32,35].

In the case of Myc-related mutations, an overexpression of Myc is linked to super-competitive behaviour that causes the cancerous cells to outproliferate their healthy neighbours. However, the same overexpression is also linked to greatly increased predisposition to apoptosis, meaning such cells may not be able to survive long enough to proliferate effectively. In [32], the authors reveal a mechanism by which such Myc^{high} mutants can survive in the presence of different, Myc^{low} mutants. These Myc^{low} mutants produce the Wnt1 transcription factor that is lacking in their Myc^{high} counterparts, and whose absence induces the affinity of Myc^{high} cells towards apoptosis.

In [32], this process is demonstrated both experimentally on *in vivo* mouse models, as well as *in silico* on a large-scale multi-valued computational model.

Furthermore, based on the perturbation response observed in the computational model, the authors identify a combination of interventions on COX2 and MEK transcription factors that together disrupt this cooperation between Myc^{low} and Myc^{high} mutants. This plausible therapeutic combination appears to be also effective in vivo.

5.2 Computational study of Myc overexpression

To study the Myc-related effects of therapeutic interventions on breast cancer, the authors of [32] design a computational model consisting of 72 variables, each variable having at least 4 levels, while several important variables extend up to 7 levels. The model is based on known literature and pathway models, and was tuned and validated using datasets from several independent studies.

The authors consider 5 variants of the final model: First, a *wildtype* (i.e., healthy) variant, then Myc^{low} and Myc^{high} variants where only one type of mutant is present in the tumour, and finally mix-Myc^{low} and mix-Myc^{high} variants, which describe the behaviour of the mutants when interacting with each other. In this case, the interaction is given as an outside assumption on the two model variants; there is no single model consisting of both Myc^{low} and Myc^{high} mutants sharing a state space.

To study the effects of possible therapeutic interventions, the authors perform knockout perturbations on the model's update functions for variables that can be plausibly influenced by known therapies. In particular, the study focuses on single and dual variable knockouts. For each such combination, the authors use `BMA` to approximate the synchronous attractors of the model. They then use the *average* of the *Proliferation* and *Apoptosis* variables in these attractors to asses the effect of the therapy on the cell fate.

In the end, they identify the combination of COX2 and MEK as a plausible therapeutic target. While this approach is certainly viable in this scenario, it has several shortcomings that we try to address in our work:

- First, while `BMA` can in theory compute the *exact* synchronous attractors of multi-valued models, the authors of [32] only rely on an approximate method, as the exact algorithm can "take an unknown or large amounts of time to find a solution". Here, we hope to demonstrate that for trap spaces, an exact method is efficient enough to analyze the models exhaustively.
- Second, while the focus on *synchronous* update scheme is to a large extent practically motivated, there are cases where plausible model behaviour can be missed due to the artificial synchronisation of variables [21]. Meanwhile, trap spaces in logical models describe behaviour that is universal regardless of the chosen update scheme [42]. It stands to reason that without any knowledge of the actual timings of *in vivo* updates, predictions based on trap spaces should generally translate to the real world more robustly as opposed to predictions based on a single specific update scheme.
- Third, the authors only consider the *average* value of Proliferation and Apoptosis within the computed attractors. While this is again a practical approximation, many complex consequences could be lost due to this simplification.

Using `trap-mvn`, we can obtain an exact interval for each of the desired variables, assessing the reliability of particular outcomes.
For example, an outcome with $Apoptosis = \{3\}$ is clearly different from $Apoptosis = \{1, 2, 3, 4, 5\}$, even though the average value is the same. In practice, even an intermittent spike in the $Apoptosis$ value could manifest as actual apoptosis in the real world. It is important to take this possibility into consideration by inspecting the full range of outcomes.

5.3 Single intervention effects on trap spaces

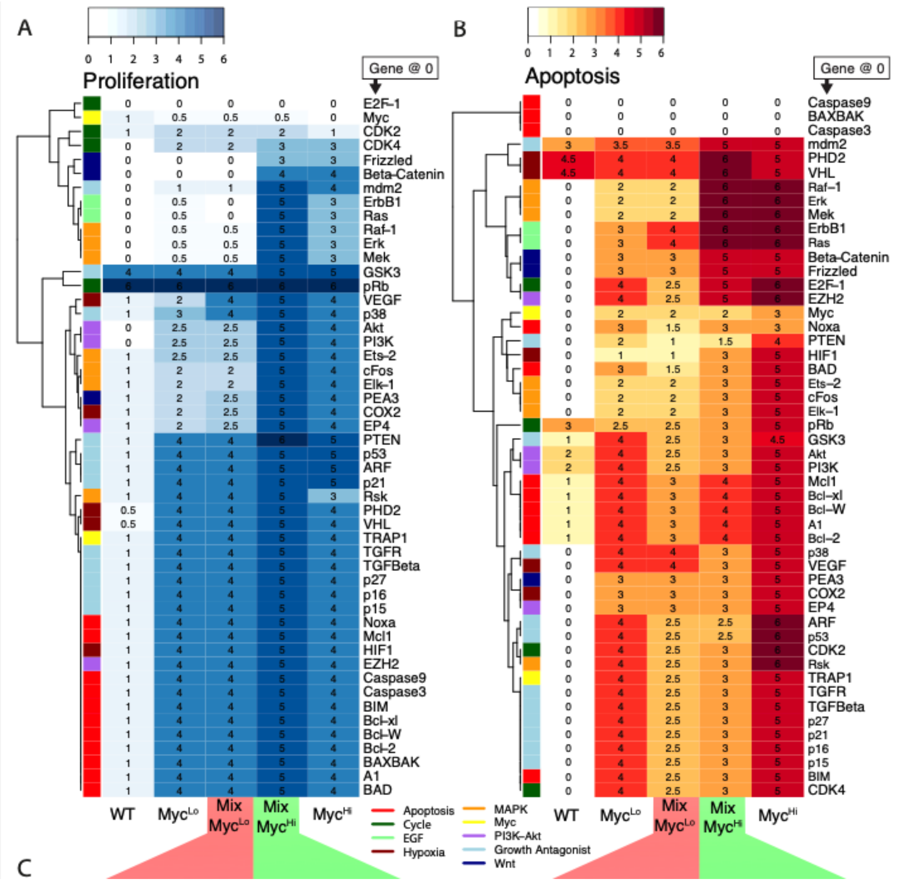

Fig. 5: Original results obtained using BMA, comparable to Tables 1 and 2.

To compare our method to the results obtained in [32], we perform a similar experiment: we take the 5 model variants used in the original paper and compute the trap spaces for the respective single and dual knockout interventions. While

Table 1: Computed trap spaces projected to the *Proliferation* variable.

Intervention	WT	Myc^{low}	mix Myc^{low}	mix Myc^{high}	Myc^{high}
E2F-1	0	0	0	0	0
CDK2	1	2	2	2	1
CDK4	0	2	2	3	3
Frizzled	0	0	0	3	3
Beta-Catenin	0	0	0	4	4
mdm2	0	0 . . . 2	0 . . . 2	5	4
ErbB1	0	0 . . . 1	0	5	3
Ras	0	0 . . . 1	0	5	3
Raf-1	0	0 . . . 1	0 . . . 1	5	3
Erk	0	0 . . . 1	**1***	5	3
Mek	0	0 . . . 1	0 . . . 1	5	3
GSK3	4	4	4	5	5
pRb	6	6	6	6	6
VEGF	1	2	4	5	4
p38	1	2 . . . 4	4	5	4
Akt	0	1 . . . 4	1 . . . 4	5	4
PI3K	0	1 . . . 4	1 . . . 4	5	4
Ets-2	1	1 . . . 4	1 . . . 4	5	4
cFos	1	0 . . . 4	0 . . . 4	5	4
Elk-1	1	0 . . . 4	0 . . . 4	5	4
PEA3	1	0 . . . 4	1 . . . 4	5	4
COX2	1	0 . . . 4	1 . . . 4	5	4
EP4	1	0 . . . 4	1 . . . 4	5	4
PTEN	1	4	4	6	5
p53	1	4	4	5	5
ARF	1	4	4	5	5
p21	1	4	4	5	5
Rsk	1	4	4	5	3
PHD2	0 . . . 1	4	4	5	4
VHL	0 . . . 1	4	4	5	4
TRAP1	1	4	4	5	4
TGFR	1	4	4	5	4
TGFBeta	1	4	4	5	4
p27	1	4	4	5	4
p16	1	4	4	5	4
p15	1	4	4	5	4
Noxa	1	4	4	5	4
Mcl1	1	4	4	5	4
HIF1	1	4	4	5	4
EZH2	1	4	4	5	4
Caspase9	1	4	4	5	4
Caspase3	1	4	4	5	4
BIM	1	4	4	5	4
Bcl-xl	1	4	4	5	4
Bcl-W	1	4	4	5	4
Bcl-2	1	4	4	5	4
BAXBAK	1	4	4	5	4
A1	1	4	4	5	4
BAD	1	4	4	5	4

Table 2: Computed trap spaces projected to the *Apoptosis* variable.

Intervention	WT	Myc^{low}	mix Myc^{low}	mix Myc^{high}	Myc^{high}
Caspase9	0	0	0	0	0
BAXBAK	0	0	0	0	0
Caspase3	0	0	0	0	0
mdm2	3	1...6	1...6	5	5
PHD2	3...6	4	4	6	5
VHL	3...6	4	4	6	5
Raf-1	0	0...4	0...4	6	6
Erk	0	0...4	**0***	6	6
Mek	0	0...4	0...4	6	6
ErbB1	0	0...6	4	6	6
Ras	0	0...6	4	6	6
Beta-Catenin	0	0...6	3	5	5
Frizzled	0	0...6	3	5	5
E2F-1	0	4	1...4	5	6
EZH2	0	4	1...4	5	6
Noxa	0	3	0...3	3	3
PTEN	0	2	1	**2***	4
HIF1	0	1	1	3	5
BAD	0	3	0...3	3	5
Ets-2	0	0...4	0...4	3	5
cFos	0	0...4	0...4	3	5
Elk-1	0	0...4	0...4	3	5
pRb	3	**4***	1...4	3	5
GSK3	1	4	1...4	3	3...6
Akt	2	2...6	2...3	3	5
PI3K	2	2...6	2...3	3	5
Mcl1	1	4	2...4	4	5
Bcl-xl	1	4	2...4	4	5
Bcl-W	1	4	2...4	4	5
A1	1	4	2...4	4	5
Bcl-2	1	4	2...4	4	5
p38	0	4	4	3	5
VEGF	0	4	4	3	5
PEA3	0	0...6	0...6	3	5
COX2	0	0...6	0...6	3	5
EP4	0	0...6	0...6	3	5
ARF	0	4	1...4	1...4	6
p53	0	4	1...4	1...4	6
CDK2	0	4	1...4	3	6
Rsk	0	4	1...4	3	6
TRAP1	0	4	1...4	3	5
TGFR	0	4	1...4	3	5
TGFBeta	0	4	1...4	3	5
p27	0	4	1...4	3	5
p21	0	4	1...4	3	5
p16	0	4	1...4	3	5
p15	0	4	1...4	3	5
BIM	0	4	1...4	3	5
CDK4	0	4	1...4	3	5

the results are in many aspects comparable to those obtained with BMA, in some cases, they paint a much more complete picture of the model's behaviour.

A knockout of a network variable is implemented by replacing its update function with a constant 0 function. Hence the knocked-out variable eventually stabilises at this value.

For the case of a single knockout, we prepared two tables (Table 1 and Table 2) that are directly comparable to Figure S5 from [32] (which is reproduced here as Figure 5). In these tables, we use the same colours as [32] to depict the resulting intensity of each variable. Note that unless states otherwise, our results are in line with the results obtained by BMA, i.e., we agree on stable values and the average attractor values obtained by BMA lie within our trap space interval.

Interpreting interval results Clearly, there are many cases where a perturbation causes a non-trivial trap space to appear, resulting in an interval instead of a fixed value for one of the considered output variables. Knowledge of these intervals is often crucial when interpreting the effectiveness of perturbations.

For example, take the case of *Apoptosis* and knockouts of Mcl1 and COX2. If we only considered the average value of *Apoptosis*, these two perturbations are equal for model mix-Myc^{low} (average value is 3). However, under Mcl1, the admissible interval is $[2, 4]$, while for COX2, it is $[0, 6]$. This means that under the Mcl1 intervention, we are guaranteed to observe *Apoptosis* value of at least 2, whereas we have no such expectation for COX2 (even though the "best case" outcome is higher; $6 > 4$).

Exact vs. approximate results There are four instances (marked with bold and *) where our method computed a tighter result than BMA. In all cases, this tighter result is actually quite different from the average value obtained through BMA. For example, in the case of *Apoptosis* and the Erk knockout, we computed value 0 whereas BMA reports an average value 2. We attribute this to the fact that in these experiments, BMA operates using an approximate algorithm. This further strengthens the case for using the exact method, especially since in this case, the complete results were obtained in just several hours.

5.4 Dual knockouts and reliable vs. opportunistic treatments

While for single knockouts, the number of possible interventions is relatively small and can be conceivably compared by hand, to pick viable treatments from dual knockouts, we need a more methodic approach. Accounting for the presence of value intervals, when comparing interventions, we consider two scenarios that we denote *reliable* and *opportunistic* interventions.

Overall, we assign a score to each perturbation, given as the sum of *Apoptosis* values across both mix-Myc^{high} and mix-Myc^{low} clones, minus the sum of *Proliferation* values across the same cells. Intuitively, our aim is to maximize *Apoptosis* while also minimizing *Proliferation*. Based on other real-world constraints, we

Table 3: Best and worst dual interventions (out of 995 tested) based on the average of their reliability and opportunity scores.

	Score			*Apoptosis*		*Proliferation*	
Intervention	Relia-bility	Oppor-tunity	WT	mix Myc^{low}	mix Myc^{high}	mix Myc^{low}	mix Myc^{high}
E2F-1 + Ras	10	10	0	4	6	0	0
ErbB1 + E2F-1	10	10	0	4	6	0	0
Beta-Catenin + E2F-1	9	9	0	3	6	0	0
Frizzled + E2F-1	9	9	0	3	6	0	0
p38 + E2F-1	9	9	0	4	5	0	0
VEGF + E2F-1	9	9	0	4	5	0	0
E2F-1 + Rsk	7	10	0	1 . . . 4	6	0	0
A1 + E2F-1	7	9	1	2 . . . 4	5	0	0
Bcl-2 + E2F-1	7	9	1	2 . . . 4	5	0	0
Bcl-W + E2F-1	7	9	1	2 . . . 4	5	0	0
			. . .				
			. . .				
VEGF + Caspase9	-9	-9	0	0	0	4	5
VHL + BAXBAK	-9	-9	0	0	0	4	5
VHL + Caspase3	-9	-9	0	0	0	4	5
VHL + Caspase9	-9	-9	0	0	0	4	5
PTEN + BAXBAK	-10	-10	0	0	0	4	6
PTEN + Caspase3	-10	-10	0	0	0	4	6
PTEN + Caspase9	-10	-10	0	0	0	4	6
BAXBAK + pRb	-12	-12	0	0	0	6	6
Caspase3 + pRb	-12	-12	0	0	0	6	6
Caspase9 + pRb	-12	-12	0	0	0	6	6

could use a different scoring function to, e.g., prioritize only *Apoptosis* (as did the authors of [32]).

Here, the *reliable* score is obtained by taking the lower bound on *Apoptosis* and upper bound on *Proliferation*. That is, we consider the "worst case" scenario admitted by the trap space. Meanwhile, the *opportunistic* score is computed based on inverse bounds (upper for *Apoptosis*, lower for *Proliferation*), which corresponds to the "best case" admitted by the model.

We then proceed to compute these metrics for every admissible dual-knockout perturbation (admissibility is judged based on druggability, as investigated in [32]). From these perturbations, we exclude cases where the perturbation causes opportunistic *Apoptosis* values in *wildtype* (i.e., healthy) cells higher than 3, as these could cause damage even to healthy cells. This leaves unique 995 perturbations.

Best and worst dual knockouts For the purposes of presentation, we sort the perturbations by the *average* of their reliability and opportunity scores. The individual scores are then defined as follows:

$$M = \{ \text{ mix-Myc}^{low}, \text{mix-Myc}^{high} \ \}$$
$$rel(I) = \sum_{m \in M} min_I(\texttt{Apop.}, m) - \sum_{m \in M} max_I(\texttt{Prolif.}, m)$$
$$opp(I) = \sum_{m \in M} max_I(\texttt{Apop.}, m) - \sum_{m \in M} min_I(\texttt{Prolif.}, m)$$

Here, $min_I(\texttt{V}, m)$ and $max_I(\texttt{V}, m)$ denote the lowest and highest value of the variable `V` for the model variant m under intervention I. In our case, `Apoptosis` contributes positively to the overall score (the treatment should maximise `Apoptosis`), while `Proliferation` contributes to the score negatively (the treatment should minimise `Proliferation`). Even though these scores are tailored for this particular model and its phenotypes, the principle is easily transferable to other models as well.

The ten best and worst perturbations are show in Table 3. As we can see, some of the best perturbations admit a non-trivial trap space for the mix-Myc^{low} model variant. However, even in this case, the reliability and opportunity scores are not vastly different.

Note that the COX2 + MEK perturbation chosen in [32] has a reliability and opportunity score equal to 5, which means it would occupy places 65-149 in Table 3 (there are 84 perturbations with the same scores). While this is not among the best scores, it is still better than 85-93% of perturbations. Furthermore, it should be noted that our screening for *viable* perturbations is rather rudimentary: in practice, some of the top perturbations might be ruled out due to factors other than the *Apoptosis* score of the *wildtype* cells (e.g., other side effects of the perturbation not captured by this model).

Distance between reliability and opportunity scores Table 3 raises a natural question: How different are the reliability and opportunity scores across all

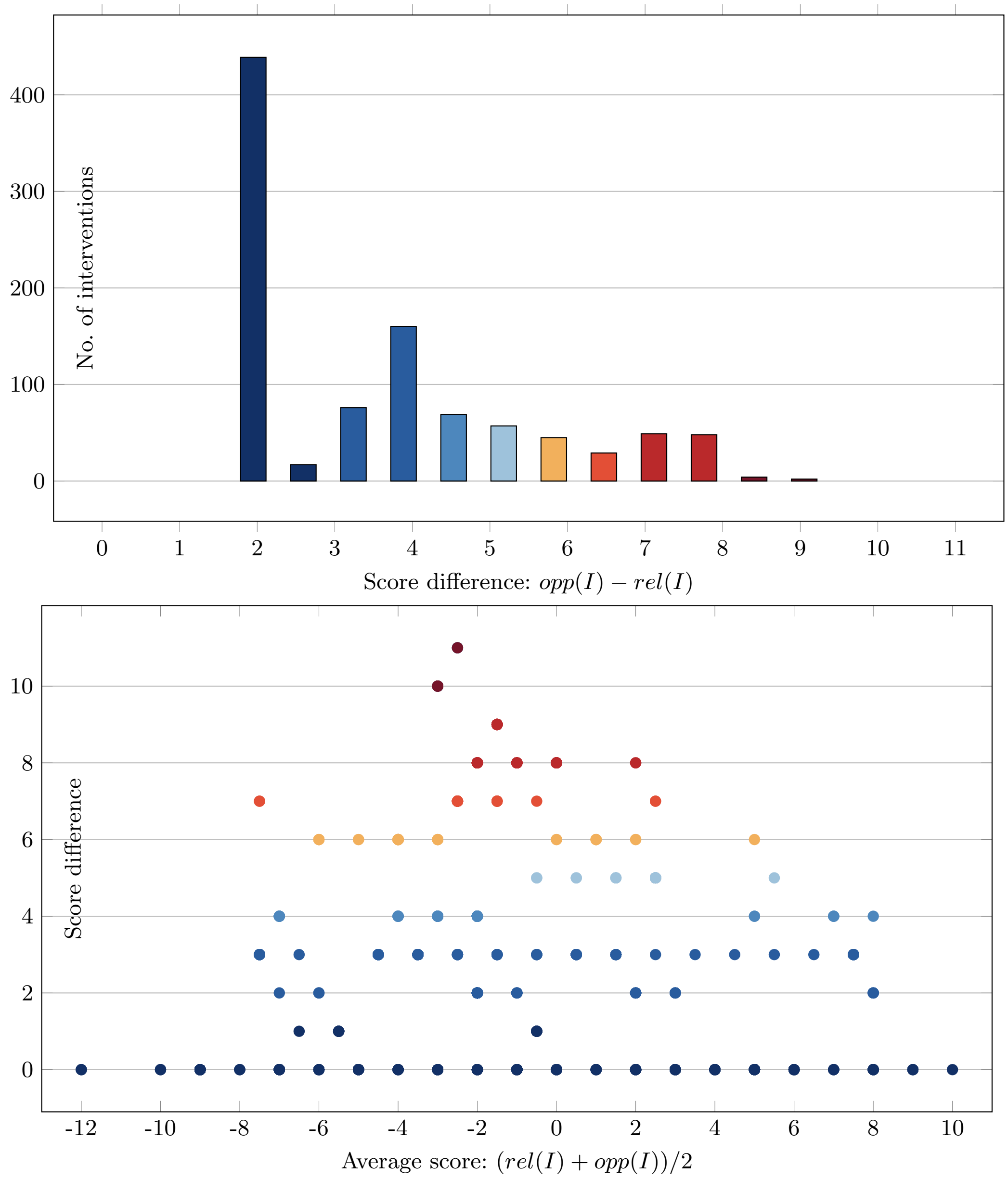

Fig. 6: Histogram of score difference values (top) and their plot in relation to the average score (bottom).

tested perturbations? To answer this question, we compute the distance between the reliability and opportunity scores of each perturbation, and plot the results as a histogram in Figure 6. As we can see, roughly half of the interventions (439/995) have the same reliability and opportunity scores.

However, for a significant portion of interventions, their opportunity and reliability scores differ substantially. In particular, for 463/995 interventions, this difference is three or more points. In these cases, it is not advisable to only rely on the average values of *Apoptosis* and *Proliferation*. Here, the model admits a wide range of behaviour and this uncertainty has to be taken into account when assessing how the perturbation can manifest in the real world.

Furthermore, as demonstrated in Figure 6, even though the scores are equivalent for the majority of the interventions, even very high differences (e.g., > 9) still appear in the dataset. Furthermore, we also plot the distribution of score differences with respect to the average score. We can see that even viable interventions (e.g., average > 4) can admit a high variability between the reliable and opportunistic metrics (up to 6).

6 Performance evaluation

In the literature, to the best of our knowledge, no work has been done so far on computing trap spaces of MVNs due to the lack of definition as well as appropriate tools. There is limited work concerning fixed-points available in [40,41,1].

To show the scalability of our approach (implemented in a Python tool called `trap-mvn`[2]), we collected several MVNs based on the known biological networks from the literature. All these models come from two sources: the `BBM` repository[3] (using the SBML-qual [7] format) and the `BMA` repository [5]. The size of these models is non-trivial and most of them have never been fully analysed. See Table 4 for the breakdown of structural properties of these models.

Experiment setup: We ran `trap-mvn` and the other existing tools on the test networks, measuring the running time. To solve the ASP problems, we used the same ASP solver Clingo [23] and the same configuration as that used by the other ASP-based methods (`mpbn`, `AN-ASP`, and `Trappist`).

Unless stated otherwise, all measured times represent an average over three runs. We have not observed significant differences between individual runs, hence we omit detailed analysis of runtime variance. Similarly, in cases where a method fails, it always fails consistently across all five runs.

All experiments ran on a desktop machine with Ryzen 5800X CPU and 128GB of RAM, Ubuntu 22.04 LTS. However, note that all tested programs only utilize one CPU core and RAM usage never exceeded 32GB.

Model formats: Since `trap-mvn` is the only tested tool which supports both `SBML` and `BMA` formats, all `BMA` models have been first translated to `SBML` (using `trap-mvn`). This translation time does not count towards the total runtime, since it would have been the same for all tools. Furthermore, for tools that operate

[2] https://github.com/giang-trinh/trap-mvn
[3] https://github.com/sybila/biodivine-boolean-models

on the Boolean network created using Van Ham encoding, the translation was facilitated by `bioLQM` [7] (into the `.bnet` format). However, this encoding step is counted towards the total tool runtime, because it is only required by some tools. Other internal encoding steps that are specific to each tool (such as the Petri net encoding within `trap-mvn`) always count towards the tool's runtime.

Table 4: Structural properties of the selected real-world models. First column indicates the source of the model (`BBM` or `BMA`). Column "name" shows a short name, including a model ID for BBM models. Columns n and k denote the number of network nodes and the sum of their domain sizes, respectively. Columns $|T_{\mathcal{M}}|$, $|T_{\mathcal{U}}|$, and $|T_{\mathcal{N}}|$ denote the number of transitions of the encoded Petri net for the general counterpart, unitary counterpart, and the Van Ham Boolean network of the unitary counterpart of each model, respectively.

	Model name	n	k	$\|T_{\mathcal{M}}\|$	$\|T_{\mathcal{U}}\|$	$\|T_{\mathcal{N}}\|$
BBM	BUDDING YEAST (146)	32	82	2643	2370	2775
BBM	AGS CELL FATE (148)	77	160	268	252	264
BBM	TCR METABOLISM (151)	111	244	951	758	862
BBM	TH1-TH2 DIFFER. (155)	65	136	238	224	235
BBM	TH DIFFER. (157)	101	204	343	337	403
BBM	YEAST CORE (159)	27	66	1558	1526	2132
BBM	IL17 DIFFER. (160)	82	174	338	324	333
BBM	MONOCYTES DIF. (161)	94	190	390	387	363
BBM	MESODERM SPEC. (167)	48	105	327	227	230
BBM	SEA URCHIN (175)	30	71	149	124	135
BBM	MYELOFIBR. ENV. (176)	40	89	226	207	209
BBM	MAST CELL (178)	47	95	112	112	115
BBM	MICROENV. CONTROL (179)	51	107	275	254	304
BBM	ALT. IN BLADDER (183)	30	65	175	152	161
BBM	BRAF TREATMENT (190)	33	70	110	103	110
BBM	SEGMENT POLARITY (192)	72	174	462	408	414
BBM	VULVAR PRECURSOR (194)	88	195	360	329	349
BBM	CTLA4 CHECKPOINT (195)	216	434	664	663	705
BBM	LYMPHOCYTE SPEC. (196)	56	117	400	385	426
BBM	ANTERIOR POST. (197)	28	84	644	454	504
BMA	MYC IN-VITRO	75	260	28403	10881	13331
BMA	MYC IN-VIVO	72	251	28323	10815	13296
BMA	METABOLISM	91	446	46984	15744	12739
BMA	LEUKAEMIA	54	161	1217	739	624
BMA	SKIN MODEL	75	375	3060	2236	2528
BMA	VPC	85	254	820	684	719

6.1 Minimal trap spaces

First, we evaluate trap-mvn on the problem of computing minimal trap spaces. We've shown (Section 3.2) that the *unitary* trap spaces of an MVN are equivalent to those of a Boolean network obtained using the Van Ham encoding [26]. As such, to evaluate the performance of trap-mvn on unitary MVNs, we can use existing state-of-the-art tools for computation of minimal trap spaces in BNs.

For this comparison, we selected mpbn [42] (version 1.7) and trappist [55]. We did not consider pyboolnet [30], because it has been shown in [55] that trappist and mpbn together outperform pyboolnet completely. For the case of *general* MVNs, there are no comparable tools that we are aware of.

We consider two related metrics of tool performance: *time to first result* (i.e. the time necessary to compute *some* minimal trap space), and *time to all results* (i.e. the time necessary to compute *all* minimal trap spaces). We configure each tool to only return the *total number* of minimal trap spaces. This eliminates the time necessary to actually print all results, which can be significant for some networks, but which can also vary based on factors like size of representation and storage speed (which are not relevant for our comparison).

Note that we separately compared the unitary trap spaces computed by all three methods and verified that they are the indeed equivalent. Also note that while the *number* of computed trap spaces is the same for the general and the unitary case, the actual computed trap spaces are often different. That is, the exact intervals of fixed variables within the computed trap spaces often differ.

Tables 5 and 6 show the full experimental results, including the number of all computed trap spaces and relative speed-up of trap-mvn compared to the relevant methods. For each competing method, we also compute the average speed-up, weighted by the runtime of the competing method. This means that benchmarks contribute to the average speed-up proportionally to their runtime ("harder" benchmarks have proportionally higher weight).

These results are visualised in Figures 7 and 8. Specifically, Figure 7 shows the relative times as a scatter plot. Here, points below the diagonal represent benchmarks where trap-mvn outperforms the competing tool. Note that the timescales in Figure 7 are logarithmic, hence even a relatively minor improvement often represents a significant speed-up. The absolute speed-ups are then analysed in Figure 8. Note that the DNF results are not included in this analysis.

Overall, we observe that trap-mvn significantly outperforms both trappist and mpbn. When enumerating all minimal trap spaces, trap-mvn is $\sim 3\times$ faster than trappist (using a weighted average), and $\sim 58\times$ faster than MPBN. Furthermore, note that MPBN could not finish large portion of the benchmarks. Finally, the time to compute the first result also enjoys similar average speed-up.

Runtime impact of ASP query density Let us now discuss the factors which contribute to the running time of each method. All three methods use ASP to some degree. However, MPBN is only applicable to locally monotonic models (21/26 of tested models). MPBN performs simplification of the Boolean functions, which is required for checking the local monotonicity and further

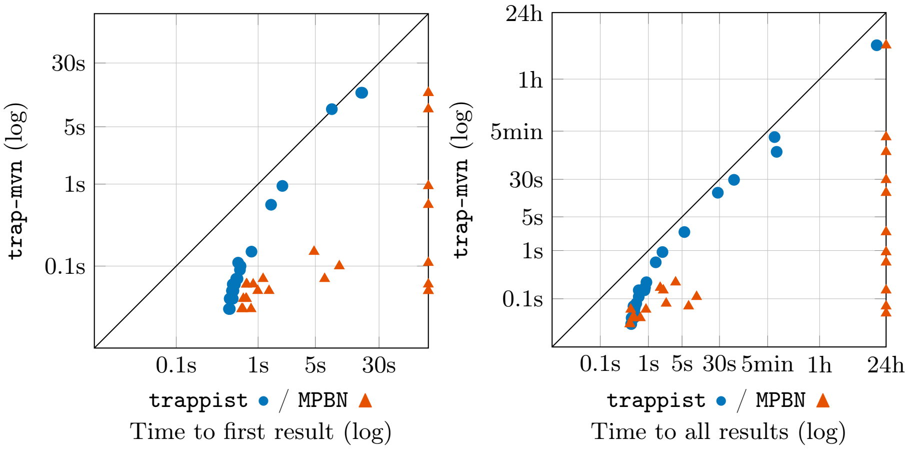

Fig. 7: Runtime comparison between `trap-mvn` and `trappist`, resp. `MPBN`, in terms of time to first result (left) and time to all results (right). Points at the right-most edge of the graph correspond to `DNF` results. Note that in both plots, the time scale is logarithmic.

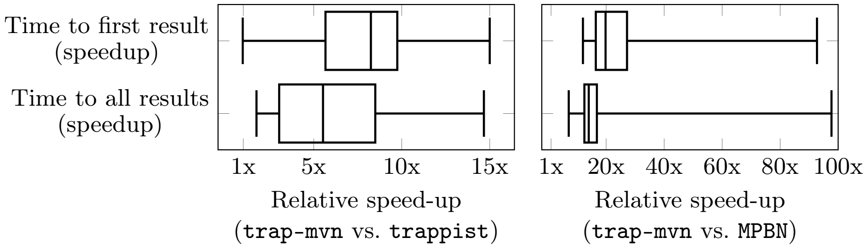

Fig. 8: Box plot summary of the relative speed-up in runtime of `trap-mvn` compared to `trappist` (left), res. `MPBN` (right).

simplifies the subsequent ASP query. However, this simplification step appears to be the bottleneck of the method, as most `DNF` results for `MPBN` happen due to this step. As such, we focus on `trappist` for the rest of this section.

Aside from the absolute number of solutions, the practical complexity of an ASP query is affected by its number of atoms and its *density*, i.e. the ratio between the number of ASP rules and atoms. For `trap-mvn` and `trappist`, the number of ASP atoms is equal to the number of Petri net places. The number of ASP rules is then equal to the number of Petri net transitions. For a general (resp. unitary) multi-valued network, its encoded Petri net has k places and $|T_{\mathcal{M}}|$ (resp. $|T_{\mathcal{U}}|$) transitions (Table 4 presents these values for our benchmark models). For the Van Ham encoded Boolean network, its encoded Petri net has $2 \times (k - n)$ places and $|T_{\mathcal{N}}|$ transitions.

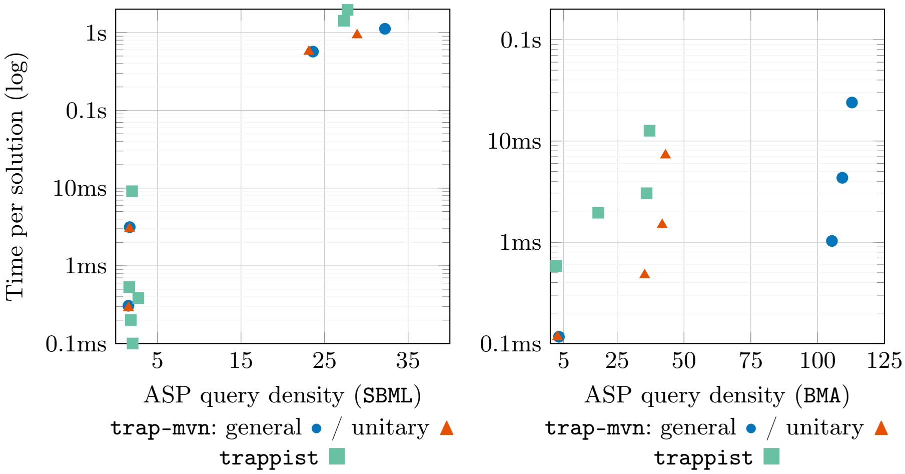

Fig. 9: Relationship between ASP query density and average time required to compute a single solution. Note that the time scale is logarithmic.

To study the relationship between query density and runtime, let us consider the following setting: As the relevant metric, we consider the time necessary to compute all results for non-trivial problems. We consider the problem to be non-trivial when (a) the runtime is more than one second, or (b) the number of trap spaces is more than 1000. For each such case, we compute the average time required to compute one solution (i.e. runtime divided by the number of trap spaces). This normalizes the runtime with respect to the solution count.

Finally, we observe that the query density of the `BMA` models appears to be generally higher than that of the `SBML` models, even for models with comparable runtime. This difference can be to some extent explained by the type of function representation employed by `BMA` and the subsequent differences in Petri net

encoding method. As such, we separate the results based on the initial model format. The results of the analysis are shown in Figure 9.

Let us note that the sample size for this analysis is not very large (12 models, 3 methods), and as such the conclusions that we can draw are limited as well. Nevertheless, Figure 9 reveals some clear trends among the employed methods.

First, all but one non-trivial model with small density (<5) are clustered in the <1ms region of the graph, while higher-density models generally require more time to produce a solution. In the `SBML` case, this manifests as a cluster of models in the 25-35 density range with time per solution close to one second. For the `BMA` models, the trend is more gradual and the results are clearly influenced by the considered method. However, if we focus on individual methods, we see that an increase in density is always accompanied by increased runtime.

To conclude, query density is clearly not the only indicator of ASP problem complexity. However, assuming we control for other relevant factors (solution count, tool/method, update scheme, model type, etc.), query density appears to be a relevant metric for comparing the complexity of two minimal trap space computation problems.

Runtime of `trap-mvn` in general and unitary cases Let us to discuss the runtime complexity of the general and unitary cases. It is quite clear to see from Tables 5 and 6 that `trap-mvn` needs more time for the general case than for the unitary case on every multi-valued network. The reason might be that the encoded Petri nets of the general and unitary MVNs have the same number of places but the former has more transitions (consequently larger density) than the latter has. This is clearly shown in Table 4. Since the other relevant factors are the same for the general and unitary multi-valued networks (the number of solutions, the tool considered, the number of places of the encoded Petri net, etc.), the difference in density appears to be the main metric for indicating the difference in runtime of `trap-mvn` in the general and unitary cases.

6.2 Maximal trap spaces

The experimental setting for the case of maximal trap spaces is similar to those for the case of minimal trap spaces. However, note that the Van Ham Boolean encoding does not preserve maximal trap spaces of neither general nor unitary MVNs. Hence, we only evaluate `trap-mvn`. Furthermore, since the absolute number of maximal trap spaces is typically small, we only measure the case of computing *all* results.

Table 7 shows the experimental results. Here, we see that the total number of maximal trap spaces is indeed small, even for networks where the number of minimal trap spaces is large. Due to this fact, the times required to compute all maximal trap spaces are comparable to the times needed to compute a single minimal trap space. Furthermore, we can observe that (as opposed to the case of minimal trap spaces), there are nine models where the number of trap spaces differs between the general and unitary interpretation of the model. Overall, `trap-mvn` performs well when enumerating maximal trap spaces.

6.3 Fixed points

Finally, we compare the runtime of `trap-mvn` on the problem of network fixed-points, which can be understood as a specialized notion of a minimal trap space where all variables are fixed. Fixed points are shared between all update schemes, hence they are the same for general, unitary and also Booleanised networks (see Section 3.2). Hence, we only consider unitary multi-valued networks hereafter.

In `trap-mvn`, we implement two methods for computing fixed-points: The first uses characterisation through PN *siphons* (the same as minimal trap spaces), the second uses characterisation through PN *deadlocks* (see Section 4.2). There are also three existing tools that support direct computation of fixed points in multi-valued networks: `GINsim` [40], `Pint` [41], and `AN-ASP` [1]. Out of these, we select `AN-ASP`, as [1] shows it to be more efficient than `GINsim` and `Pint`.

Note that `AN-ASP` requires an automata network as input. Therefore, we use `bioLQM` [7] to convert an `SBML` multi-valued network into an equivalent automata network. This conversion counts towards the total runtime of `AN-ASP`.

In addition, we can also consider tools that operate on multi-valued networks Booleanised through the Van Ham encoding (see Theorem 4). Here, we again compare to `trappist` [55] and `MPBN` [42] as state of the art methods. Note that, similar to `trap-mvn`, `trappist` also implements two fixed-point computation methods based on PN siphons and deadlocks.

In Section 6.1, we saw that the time to first result appears to be a suitable metric to test method performance (i.e. speed-up results were similar for time to first result and time to all results). As such, to simplify the tool comparison, we only consider time to first result in our fixed point analysis.

Table 8 shows the experimental results. Note that `MPBN` times out on the same networks as when testing the minimum trap spaces, due to the previously outlined bottleneck in model simplification. Overall, its running time is comparable to the case of minimum trap spaces. As expected, the running times of the siphon-based methods of `trap-mvn` and `trappist` are also comparable to the case of minimal trap spaces. However, the deadlock-based methods are in all instances faster than the siphon-based methods. In fact, using the same weighted average metric as before, the deadlock method of `trap-mvn` is $\sim 2.6\times$ faster than the siphon method. Comparing the deadlock-based methods of `trap-mvn` and `trappist`, we see that on average, the `trap-mvn` method is faster, but on hard benchmarks, the improvement is not significant.

However, observe that `AN-ASP` outperforms both `trap-mvn` and `trappist` ($\sim 1.3\times$ weighted average speed-up). This is to be expected, as `AN-ASP` is optimised solely for fixed-point computation, and it highlights the need for specialised methods for similar but related problems.

7 Conclusion

In this article, we have generalized the concept of trap spaces in Boolean networks to that in multi-valued networks. Then, we have explored and proved

several properties of trap spaces in multi-valued networks as well as shown the theoretical applications of trap spaces in the analysis and control of multi-valued networks. Next, we have made a connection between trap spaces of a multi-valued network and siphons of its Petri net encoding. A very first theoretical application of this connection is to provide another way to prove the independence of trap spaces of a multi-valued network to the update scheme. We believe that this connection also provides a useful tool for exploring and proving further theoretical results of trap spaces in multi-valued networks. In computational aspects, from this connection, we have proposed and implemented a new method based on answer set programming [23] for computing different types of trap spaces of a multi-valued network and a special method for computing fixed points. We have shown the applicability of our methods via a realistic case study and evaluated their time efficiency by conducting experiments on real-world models collected from the literature.

The experimental results show that our methods scale well with the network size and in particular they can handle large-scale models. The indirect approach (i.e., building a Boolean mapping of a multi-valued network and applying the methods developed for Boolean networks) is only applicable for the case of fixed points and the case of minimal trap spaces of unitary multi-valued networks. For these cases, the direct approach (i.e., our proposed methods) outperforms the indirect one, which confirms our expectation about direct and efficient analysis methods for multi-valued networks.

In addition, there are possibly other methods for computing minimal/maximal conflict-free siphons in Petri nets, like SAT/MaxSAT approaches [39]. Although these approaches do not directly support the minimal/maximal conflict-free siphon computation now, we plan to investigate them in the future. They could replace our ASP method if they outperform it. However, the current method appears to already perform very well even on the most large and complex models we have considered.

Trap spaces only capture static behavior of a multi-valued network, whereas attractors capture the more complex dynamical behavior. Hence, we plan to attack the attractor detection in multi-valued networks as well as explore more theoretical results that can contribute to the theory of multi-valued networks. Exploiting the relation between attractors and trap spaces of multi-valued networks is a potentially promising solution.

Finally, we plan to develop efficient control methods for biological systems modeled by multi-valued networks because the control problem is crucial in systems biology and can be seen as the sequel of the trap space or attractor analysis [19]. Extending the trap spaces-based methods [19,45,12] for Boolean networks is a potential direction. Furthermore, we also want to apply our developed methods to specific biological problems, which requires a closed collaboration with biologists.

References

1. Abdallah, E.B., Folschette, M., Roux, O.F., Magnin, M.: ASP-based method for the enumeration of attractors in non-deterministic synchronous and asynchronous multi-valued networks. Algorithms Mol. Biol. **12**(1), 20:1–20:23 (2017)
2. Akutsu, T.: Algorithms for analysis, inference, and control of Boolean networks. World Scientific (2018)
3. Bahar, R.I., Frohm, E.A., Gaona, C.M., Hachtel, G.D., Macii, E., Pardo, A., Somenzi, F.: Algebric decision diagrams and their applications. Formal Methods Syst. Des. **10**(2), 171–206 (1997)
4. Basser-Ravitz, E., Darbar, A., Chifman, J.: Cyclic attractors of nonexpanding q-ary networks. J. Math. Biol. **85**(5), 1–31 (Oct 2022)
5. Benque, D., Bourton, S., Cockerton, C., Cook, B., Fisher, J., Ishtiaq, S., Piterman, N., Taylor, A., Vardi, M.Y.: BMA: Visual tool for modeling and analyzing biological networks. In: Computer Aided Verification, pp. 686–692. Springer Berlin Heidelberg (2012)
6. Bryant, R.E.: Graph-based algorithms for Boolean function manipulation. IEEE Trans. Comput. **35**(8), 677–691 (1986)
7. Chaouiya, C., Bérenguier, D., Keating, S.M., Naldi, A., et al.: SBML qualitative models: a model representation format and infrastructure to foster interactions between qualitative modelling formalisms and tools. BMC Syst. Biol. **7**, 135 (2013)
8. Chaouiya, C., Naldi, A., Remy, E., Thieffry, D.: Petri net representation of multi-valued logical regulatory graphs. Nat. Comput. **10**(2), 727–750 (2011)
9. Chaouiya, C., Remy, E., Ruet, P., Thieffry, D.: Qualitative modelling of genetic networks: From logical regulatory graphs to standard Petri nets. In: Applications and Theory of Petri Nets 2004, 25th International Conference, ICATPN 2004, Bologna, Italy, June 21-25, 2004, Proceedings. Lecture Notes in Computer Science, vol. 3099, pp. 137–156. Springer (2004)
10. Chatain, T., Haar, S., Jezequel, L., Paulevé, L., Schwoon, S.: Characterization of reachable attractors using Petri net unfoldings. In: Computational Methods in Systems Biology - 12th International Conference, CMSB 2014, Manchester, UK, November 17-19, 2014, Proceedings. Lecture Notes in Computer Science, vol. 8859, pp. 129–142. Springer (2014)
11. Chifman, J., Arat, S., Deng, Z., Lemler, E., Pino, J.C., Harris, L.A., Kochen, M.A., Lopez, C.F., Akman, S.A., Torti, F.M., Torti, S.V., Laubenbacher, R.: Activated oncogenic pathway modifies iron network in breast epithelial cells: A dynamic modeling perspective. PLoS Comput. Biol. **13**(2), e1005352 (Feb 2017)
12. Cifuentes-Fontanals, L., Tonello, E., Siebert, H.: Node and edge control strategy identification via trap spaces in Boolean networks. arXiv preprint (2022)
13. Corral-Jara, K.F., Chauvin, C., Abou-Jaoudé, W., Grandclaudon, M., Naldi, A., Soumelis, V., Thieffry, D.: Interplay between SMAD2 and STAT5A is a critical determinant of IL-17A/IL-17F differential expression. Mol. Biomed. **2**(1), 1–16 (2021)
14. Delaplace, F., Ivanov, S.: Bisimilar Booleanization of multivalued networks. Biosyst. **197**, 104205 (2020)
15. Didier, G., Remy, E., Chaouiya, C.: Mapping multivalued onto Boolean dynamics. J. Theor. Biol. **270**(1), 177–184 (Feb 2011)
16. Dinh, K., Wang, Q.: A probabilistic Boolean model on hair follicle cell fate regulation by TGF-β. Biophys. J. **121**(13), 2638–2652 (Jul 2022). https://doi.org/10.1016/j.bpj.2022.05.035

17. Fauré, A., Kaji, S.: A circuit-preserving mapping from multilevel to Boolean dynamics. J. Theor. Biol. **440**, 71–79 (Mar 2018)
18. Floc'Hlay, S., Molina, M.D., Hernandez, C., Haillot, E., Thomas-Chollier, M., Lepage, T., Thieffry, D.: Deciphering and modelling the TGF-β signalling interplays specifying the dorsal-ventral axis of the sea urchin embryo. Dev. **148**(2), dev189944 (2021). https://doi.org/10.1242/dev.189944
19. Fontanals, L.C., Tonello, E., Siebert, H.: Control strategy identification via trap spaces in Boolean networks. In: Computational Methods in Systems Biology - 18th International Conference, CMSB 2020, Konstanz, Germany, September 23-25, 2020, Proceedings. Lecture Notes in Computer Science, vol. 12314, pp. 159–175. Springer (2020)
20. Gan, X., Albert, R.: General method to find the attractors of discrete dynamic models of biological systems. Phys. Rev. E **97**, 042308–042325 (Apr 2018)
21. Garg, A., Cara, A.D., Xenarios, I., Mendoza, L., Micheli, G.D.: Synchronous versus asynchronous modeling of gene regulatory networks. Bioinform. **24**(17), 1917–1925 (2008)
22. Garg, A., Mendoza, L., Xenarios, I., DeMicheli, G.: Modeling of multiple valued gene regulatory networks. In: 2007 29th Annual International Conference of the IEEE Engineering in Medicine and Biology Society. pp. 1398–1404. IEEE (Aug 2007)
23. Gebser, M., Kaufmann, B., Kaminski, R., Ostrowski, M., Schaub, T., Schneider, M.: Potassco: The Potsdam answer set solving collection. AI Commun. **24**(2), 107–124 (2011)
24. Gerlinger, M., Rowan, A.J., Horswell, S., Larkin, J., Endesfelder, D., Gronroos, E., Martinez, P., Matthews, N., Stewart, A., Tarpey, P., et al.: Intratumor heterogeneity and branched evolution revealed by multiregion sequencing. N. Engl. J. Med. **366**, 883–892 (2012)
25. Giang, T.V., Akutsu, T., Hiraishi, K.: An FVS-based approach to attractor detection in asynchronous random Boolean networks. IEEE ACM Trans. Comput. Biol. Bioinform. **19**(2), 806–818 (2022). https://doi.org/10.1109/TCBB.2020.3028862
26. Ham, P.V.: How to deal with variables with more than two levels. In: Lecture Notes in Biomathematics, pp. 326–343. Springer Berlin Heidelberg (1979)
27. Heselmeyer-Haddad, K., Garcia, L.Y.B., Bradley, A., Ortiz-Melendez, C., Lee, W.J., Christensen, R., Prindiville, S.A., Calzone, K.A., Soballe, P.W., Hu, Y., et al.: Single-cell genetic analysis of ductal carcinoma in situ and invasive breast cancer reveals enormous tumor heterogeneity yet conserved genomic imbalances and gain of myc during progression. Am. J. Pathol. **181**(5), 1807–1822 (2012)
28. Kitano, H.: Cancer as a robust system: implications for anticancer therapy. Nat. Rev. Cancer **4**(3), 227–235 (Mar 2004). https://doi.org/10.1038/nrc1300
29. Klarner, H., Bockmayr, A., Siebert, H.: Computing maximal and minimal trap spaces of Boolean networks. Nat. Comput. **14**(4), 535–544 (2015)
30. Klarner, H., Streck, A., Siebert, H.: PyBoolNet: a python package for the generation, analysis and visualization of Boolean networks. Bioinform. **33**(5), 770–772 (2017)
31. Kortlever, R.M., Sodir, N.M., Wilson, C.H., Burkhart, D.L., Pellegrinet, L., Swigart, L.B., Littlewood, T.D., Evan, G.I.: Myc cooperates with Ras by programming inflammation and immune suppression. Cell **171**(6), 1301–1315 (2017)
32. Kreuzaler, P., Clarke, M.A., Brown, E.J., Wilson, C.H., Kortlever, R.M., Piterman, N., Littlewood, T., Evan, G.I., Fisher, J.: Heterogeneity of Myc expression in breast cancer exposes pharmacological vulnerabilities revealed through executable mechanistic modeling. Proc. Natl. Acad. Sci. U.S.A. **116**(44), 22399–22408 (2019)

33. Liu, G., Barkaoui, K.: A survey of siphons in Petri nets. Inf. Sci. **363**, 198–220 (2016)
34. Lodish, H.F., Berk, A., Zipursky, S.L., Matsudaira, P., Baltimore, D., Darnell, J., et al.: Molecular cell biology, vol. 4. WH Freeman New York (2000)
35. Marusyk, A., Tabassum, D.P., Altrock, P.M., Almendro, V., Michor, F., Polyak, K.: Non-cell-autonomous driving of tumour growth supports sub-clonal heterogeneity. Nature **514**(7520), 54–58 (2014)
36. Mendik, P., Kerestély, M., Kamp, S., Deritei, D., Kunšič, N., Vassy, Z., Csermely, P., Veres, D.V.: Translocating proteins compartment-specifically alter the fate of epithelial-mesenchymal transition in a compartmentalized Boolean network model. npj Syst. Biol. Appl. **8**(1), 19 (Jun 2022). https://doi.org/10.1038/s41540-022-00228-7, https://doi.org/10.1038/s41540-022-00228-7
37. Murata, T.: Petri nets: Properties, analysis and applications. Proc. IEEE **77**(4), 541–580 (Apr 1989)
38. Mushthofa, M., Schockaert, S., Hung, L., Marchal, K., Cock, M.D.: Modeling multi-valued biological interaction networks using fuzzy answer set programming. Fuzzy Sets Syst. **345**, 63–82 (2018)
39. Nabli, F., Martinez, T., Fages, F., Soliman, S.: On enumerating minimal siphons in Petri nets using CLP and SAT solvers: theoretical and practical complexity. Constraints An Int. J. **21**(2), 251–276 (2016)
40. Naldi, A., Thieffry, D., Chaouiya, C.: Decision diagrams for the representation and analysis of logical models of genetic networks. In: Computational Methods in Systems Biology, International Conference, CMSB 2007, Edinburgh, Scotland, September 20-21, 2007, Proceedings. Lecture Notes in Computer Science, vol. 4695, pp. 233–247. Springer (2007)
41. Paulevé, L.: Pint: A static analyzer for transient dynamics of qualitative networks with IPython interface. In: Computational Methods in Systems Biology - 15th International Conference, CMSB 2017, Darmstadt, Germany, September 27-29, 2017, Proceedings. Lecture Notes in Computer Science, vol. 10545, pp. 309–316. Springer (2017)
42. Paulevé, L., Kolčák, J., Chatain, T., Haar, S.: Reconciling qualitative, abstract, and scalable modeling of biological networks. Nat. Commun. **11**(1), 1–7 (Aug 2020)
43. Remy, E., Rebouissou, S., Chaouiya, C., Zinovyev, A., Radvanyi, F., Calzone, L.: A modeling approach to explain mutually exclusive and co-occurring genetic alterations in bladder tumorigenesis. Cancer Research **75**(19), 4042–4052 (Sep 2015)
44. Richard, A.: Negative circuits and sustained oscillations in asynchronous automata networks. Adv. Appl. Math. **44**(4), 378–392 (2010)
45. Rozum, J.C., Deritei, D., Park, K.H., Gómez Tejeda Zañudo, J., Albert, R.: pystablemotifs: Python library for attractor identification and control in Boolean networks. Bioinform. **38**(5), 1465–1466 (2021)
46. Rozum, J.C., Zañudo, J.G.T., Gan, X., Deritei, D., Albert, R.: Parity and time reversal elucidate both decision-making in empirical models and attractor scaling in critical Boolean networks. Sci. Adv. **7**(29), eabf8124 (Jul 2021). https://doi.org/10.1126/sciadv.abf8124
47. Schaub, M.A., Henzinger, T.A., Fisher, J.: Qualitative networks: a symbolic approach to analyze biological signaling networks. BMC Syst. Biol. **1**(1), 1–21 (2007)
48. Schlatter, R., Schmich, K., Vizcarra, I.A., Scheurich, P., Sauter, T., Borner, C., Ederer, M., Merfort, I., Sawodny, O.: ON/OFF and beyond - A Boolean model of apoptosis. PLoS Comput. Biol. **5**(12) (2009). https://doi.org/10.1371/journal.pcbi.1000595

49. Schwab, J.D., Kühlwein, S.D., Ikonomi, N., Kühl, M., Kestler, H.A.: Concepts in Boolean network modeling: What do they all mean? Comput. Struct. Biotechnol. J. **18**, 571–582 (2020)
50. Steinway, S.N., Zañudo, J.G.T., Michel, P.J., Feith, D.J., Loughran, T.P., Albert, R.: Combinatorial interventions inhibit TGFβ-driven epithelial-to-mesenchymal transition and support hybrid cellular phenotypes. npj Syst. Biol. Appl. **1**(1), 1–12 (Nov 2015). https://doi.org/10.1038/npjsba.2015.14
51. Sun, Z., Jin, X., Albert, R., Assmann, S.M.: Multi-level modeling of light-induced stomatal opening offers new insights into its regulation by drought. PLoS Comput. Biol. **10**(11), e1003930 (Nov 2014). https://doi.org/10.1371/journal.pcbi.1003930
52. Sun, Z., Jin, X., Albert, R., Assmann, S.M.: Multi-level modeling of light-induced stomatal opening offers new insights into its regulation by drought. PLoS Comput. Biol. **10**(11), e1003930 (2014). https://doi.org/10.1371/journal.pcbi.1003930
53. Thieffry, D., Thomas, R.: Dynamical behaviour of biological regulatory networks—II. immunity control in bacteriophage lambda. Bull. Math. Biol. **57**(2), 277–297 (Mar 1995)
54. Thomas, R.: Regulatory networks seen as asynchronous automata: a logical description. J. Theor. Biol. **153**(1), 1–23 (1991)
55. Trinh, V., Benhamou, B., Hiraishi, K., Soliman, S.: Minimal trap spaces of logical models are maximal siphons of their Petri net encoding. In: Computational Methods in Systems Biology - 20th International Conference, CMSB 2022, Bucharest, Romania, September 14-16, 2022, Proceedings. Lecture Notes in Computer Science, vol. 13447, pp. 158–176. Springer (2022)
56. Trinh, V., Hiraishi, K., Benhamou, B.: Computing attractors of large-scale asynchronous Boolean networks using minimal trap spaces. In: ACM International Conference on Bioinformatics, Computational Biology and Health Informatics. pp. 13:1–13:10. ACM (2022). https://doi.org/10.1145/3535508.3545520
57. Tsirvouli, E., Ashcroft, F., Johansen, B., Kuiper, M.: Logical and experimental modeling of cytokine and eicosanoid signaling in psoriatic keratinocytes. iScience **24**(12), 103451 (2021). https://doi.org/10.1016/j.isci.2021.103451
58. Valverde, J.C., Mortveit, H.S., Gershenson, C., Shi, Y.: Boolean networks and their applications in science and engineering. Complex. **2020**, 6183798:1–6183798:3 (2020)
59. Vita, M., Henriksson, M.: The Myc oncoprotein as a therapeutic target for human cancer. Semin. Cancer Biol. **16**(4), 318–330 (Aug 2006)
60. Wollbold, J., Jaster, R., Müller, S., Rateitschak, K., Wolkenhauer, O.: Anti-inflammatory effects of reactive oxygen species - a multi-valued logical model validated by formal concept analysis. BMC Syst. Biol. **8**, 1–20 (2014). https://doi.org/10.1186/s12918-014-0101-7
61. Wooten, D.J., Zañudo, J.G.T., Murrugarra, D., Perry, A.M., Dongari-Bagtzoglou, A., Laubenbacher, R.C., Nobile, C.J., Albert, R.: Mathematical modeling of the Candida albicans yeast to hyphal transition reveals novel control strategies. PLoS Comput. Biol. **17**(3), e1008690 (2021). https://doi.org/10.1371/journal.pcbi.1008690
62. Zañudo, J.G.T., Albert, R.: Cell fate reprogramming by control of intracellular network dynamics. PLoS Comput. Biol. **11**(4), e1004193 (2015). https://doi.org/10.1371/journal.pcbi.1004193, https://doi.org/10.1371/journal.pcbi.1004193

A Van Ham encoding preserves unitary trap spaces

Recall that a *sub-space* of a multi-valued network is a mapping s which assigns each $v_i \in \mathcal{V}$ a subset of its domain set K_i, i.e., $s(v_i) \subseteq K_i$.

We call a space *continuous* if every $s(v_i)$ is an integer interval: $s(v_i) = [k, l]$ for some $k, l \in \mathbb{N}_0$.

The Van Ham encoding of a multi-valued network represents each variable v_i using $|K_i| - 1$ Boolean variables (here also called *bits*) which we denote $b_i^{(1)}, \dots b_i^{(m_i)}$ (with m_i being the maximal value of v_i). In this encoding, a value $k \in K_i$ corresponds to a valuation of the Boolean variables where $b_i^{(j)} = \mathit{true}$ if and only if $j \leq k$.

For convenience, we will denote this as strings of 1/0 symbols. For example, 11100 denotes value 3 of a variable where $|K_i| = [0, 5]$. Clearly, only a small fraction of such Boolean valuations represent a valid integer value. We refer to the rest as *invalid* values, or invalid encodings (e.g., 11010 represents an invalid value).

The resulting Boolean network in Van Ham encoding then has the following update functions $f_{b_i^{(j)}}(x) = (b_i^{(j-1)} \wedge f_{i,j}(x)) \vee b_i^{(j+1)}$ (assuming $b_i^{(0)} = \mathit{true}$ and $b_i^{(|K_i|)} = \mathit{false}$). Here, $f_{i,j}$ is a function which is true when variable v_i can transition to level j (i.e., $f_{i,j}(x) = 1 \Leftrightarrow F_i(x) \geq j$, F_i being the original multi-valued function).

This encoding preserves two important properties:

– Bit $b^{(j)}$ can become active only when $b^{(j-1)}$ is active.
– Bit $b^{(j)}$ cannot become inactive while $b^{(j+1)}$ is active.

In terms of the original paper by Van Ham, these are the *continuity conditions* of the encoding. Also, later it has been shown that if restricted to the valid states, the asynchronous STG of Van Ham encoding is isomorphic to the asynchronous STG of the original network.

Finally, observe that due to the unitary semantics, every $f_{i,j}(x)$ can be constructed such that if it depends on the value of v_i, it only depends on the bits $b_i^{(j-1)}$, $b_i^{(j)}$, and $b_i^{(j+1)}$ (essentially, $v_i \geq (j-1)$, $v_i \geq j$, and $v_i \geq (j+1)$).

Let us now observe several useful properties of the Van Ham encoding:

Theorem 8. *Any state with an invalid value of variable v_i can reach a state with a valid value of v_i.*

Proof: Since the value of v_i is encoded incorrectly, there must be a bit $b_i^{(j)} = 0$ such that $b_i^{(j+1)} = 1$. Based on the construction of $f_{b_i^{(j)}}$, we now have that $f_{b_i^{(j)}}(x) = 1$ regardless of x. Hence the encoded Boolean network can transition to a state with $b_i^{(j)} = 1$. Such state either has a valid value of v_i, or the process is repeated (up to $|K_i| - 2$ times) until the value is finally valid.

Theorem 9. *No state with an invalid encoding of v_i can be reached from a state with a valid encoding of v_i.*

Proof: Follows from the continuity conditions.

Corollary 1. *Any trap space of a Van Ham encoding contains at least one valid state.*

Theorem 10. *Any continuous trap space of the original network is a trap space of the Van Ham encoded network.*

Proof: For a contradiction, assume that this is not true and there is an asynchronous transition leaving the encoded trap space.

First, this transition cannot originate in any properly encoded state. The asynchronous STG is isomorphic to the one of the original network. Hence such transition must exist in the original network as well and the space is not a trap space.

As such, the transition originates in an invalid state and modifies a variable v_i which belongs to an interval $[k, l]$ (defined by the trap space). This means that in the Boolean encoding, bits $b_i^{(k+1)}, \ldots, b_i^{(l)}$ are "free". Now, suppose the transition modifies a bit $j \in [0, m_i]$.

- If $j \in [1, k-1]$, then the bit cannot be modified due to continuity (bit $b_i^{(j+1)}$ is 1).
- If $j \in [l+2, m_i]$, then the bit cannot be modified due to continuity (bit $b_i^{(j-1)}$ is 0).
- If $j \in [k+1, l]$, then modifying the bit will not leave the trap space.
- This leaves bits $j = k$ and $j = l + 1$, with the assumption that the invalid state still satisfies the continuity requirements (otherwise the transition is impossible). First, let's take $j = k$, meaning the transition sets the value of $b_i^{(k)}$ from 1 to 0. This also implies $b_i^{(k+1)} = 0$. Hence $f_{i,k}(x) = 0$. Due to our new assumption (above), we know that $f_{i,k}$ only depends on $b_i^{(k-1)}$, $b_i^{(k)}$, and $b_i^{(k+1)}$. We thus also know that $f_{i,k}(x[v_i = k]) = 0$, where $x[v_i = k]$ is the copy of x with a correclty encoded value of $v_i = k$. The case of $j = l + 1$ is then symmetric.

Consequently, there can be no such invalid transition and the trap space of the original network is also a trap space of the Van Ham encoding.

Note that if the original space is minimal, the new space is minimal as well: Assume there was a subspace that was a trap space. Then this subspace must contain a valid state, and hence (by the isomorphism assumption), the valid states of the subspace must form a trap space in the original network, constradicting the minimality of the original space.

Table 5: Time to obtain the first result for minimal trap space computation. All times are in seconds. Where relevant, we give speed-up of `trap-mvn` compared to the competing tool, and the average speed-up weighted by the runtime of the competing tool. `DNF` denotes time-out of one hour. `NM` are non-monotonic networks which cannot be processed using `MPBN`.

	general	unitary				
Model name	trap mvn	trap mvn	trappist	speed-up trappist	MPBN	speed-up MPBN
BUDDING YEAST (146)	1.06	0.95	1.98	2.0x	DNF	-
AGS CELL FATE (148)	0.04	0.04	0.49	12.2x	0.71	17.7x
TCR METABOLISM (151)	0.12	0.10	0.61	6.1x	9.81	98.1x
TH1-TH2 DIFFER. (155)	0.05	0.05	0.48	9.6x	NM	-
TH DIFFER. (157)	0.06	0.06	0.50	8.3x	0.72	12.0x
YEAST CORE (159)	0.58	0.56	1.44	2.5x	DNF	-
IL17 DIFFER. (160)	0.06	0.06	0.52	8.6x	0.73	12.1x
MONOCYTES DIF. (161)	0.06	0.06	0.52	8.6x	0.72	12.0x
MESODERM SPEC. (167)	0.06	0.05	0.49	9.8x	0.99	19.8x
SEA URCHIN (175)	0.03	0.03	0.45	15.0x	0.82	27.3x
MYELOFIBR. ENV. (176)	0.04	0.04	0.45	11.2x	0.72	18.0x
MAST CELL (178)	0.03	0.03	0.44	14.6x	0.63	21.0x
MICROENV. CONTROL (179)	0.05	0.05	0.50	10.0x	1.36	27.2x
ALT. IN BLADDER (183)	0.04	0.04	0.45	11.2x	0.66	16.5x
BRAF TREATMENT (190)	0.03	0.03	0.45	15.0x	0.65	21.6x
SEGMENT POLARITY (192)	0.06	0.06	0.50	8.3x	0.87	14.5x
VULVAR PRECURSOR (194)	0.05	0.05	0.48	9.6x	NM	-
CTLA4 CHECKPOINT (195)	0.09	0.09	0.60	6.6x	NM	-
LYMPHOCYTE SPEC. (196)	0.07	0.06	0.49	8.1x	NM	-
ANTERIOR POST. (197)	0.09	0.07	0.54	7.7x	6.49	92.7x
MYC IN-VITRO	45.28	13.01	18.67	1.4x	DNF	-
MYC IN-VIVO	45.50	13.02	18.17	1.4x	DNF	-
METABOLISM	0.16	0.11	0.57	5.1x	NM	-
LEUKAEMIA	32.29	8.21	7.95	0.9x	DNF	-
SKIN MODEL	0.22	0.15	0.83	5.5x	4.82	32.1x
VPC	0.08	0.07	0.56	8.0x	1.15	16.4x
		Average weighted speed-up:		2.8x		60.2x

Table 6: Time to obtain all minimal trap spaces. All times are in seconds and $|M|$ denotes the number of trap spaces. Where relevant, we give speed-up of **trap-mvn** compared to the competing tool, and the average speed-up weighted by the runtime of the competing tool. DNF denotes time-out of 24 hours. NM are non-monotonic networks which cannot be processed using MPBN.

	general		unitary					
Model name	$\|M\|$	trap mvn	$\|M\|$	trap mvn	trappist	speed-up trappist	MPBN	speed-up MPBN
BUDDING YEAST (146)	1	1.12	1	0.93	1.96	2.1x	DNF	-
AGS CELL FATE (148)	1	0.04	1	0.04	0.5	12.5x	0.47	11.7x
TCR METABOLISM (151)	9	0.12	9	0.11	0.63	5.7x	10.03	91.1x
TH1-TH2 DIFFER. (155)	68100	2.29	68100	2.42	5.59	2.3x	NM	-
TH DIFFER. (157)	6063664	19074.11	6063664	18165.82	55221.59	3.0x	DNF	-
YEAST CORE (159)	1	0.57	1	0.57	1.42	2.4x	DNF	-
IL17 DIFFER. (160)	4120	0.16	4120	0.15	0.83	5.5x	2.04	13.6x
MONOCYTES DIF. (161)	4	0.06	4	0.06	0.51	8.5x	0.43	7.1x
MESODERM SPEC. (167)	9088	0.24	9088	0.22	0.91	4.1x	3.67	16.6x
SEA URCHIN (175)	654	0.04	654	0.04	0.45	11.2x	0.68	17.0x
MYELOFIBR. ENV. (176)	4	0.04	4	0.04	0.48	12.0x	0.49	12.2x
MAST CELL (178)	19	0.03	19	0.03	0.44	14.6x	0.41	13.6x
MICROENV. CONTROL (179)	1452	0.09	1452	0.08	0.56	7.0x	2.34	29.2x
ALT. IN BLADDER (183)	25	0.04	25	0.04	0.45	11.2x	0.51	12.7x
BRAF TREATMENT (190)	32	0.03	32	0.03	0.44	14.6x	0.42	14.0x
SEGMENT POLARITY (192)	65	0.07	65	0.06	0.51	8.5x	0.89	14.8x
VULVAR PRECURSOR (194)	39	0.05	39	0.05	0.51	10.2x	NM	-
CTLA4 CHECKPOINT (195)	781216	239.43	781216	225.75	416.79	1.8x	NM	-
LYMPHOCYTE SPEC. (196)	28	0.07	28	0.07	0.49	7.0x	NM	-
ANTERIOR POST. (197)	1	0.09	1	0.07	0.53	7.5x	6.84	97.7x
MYC IN-VITRO	19707	85.34	19707	29.29	60.03	2.0x	DNF	-
MYC IN-VIVO	2187	52.45	2187	15.83	27.65	1.7x	DNF	-
METABOLISM	235160	242.27	235160	111.58	461.03	4.1x	NM	-
LEUKAEMIA	729	0.21	729	0.15	0.63	4.2x	DNF	-
SKIN MODEL	10000000+	DNF	10000000+	DNF	DNF	-	DNF	-
VPC	1458	0.17	1458	0.17	0.85	5.0x	1.76	10.3x
					Average weighted speed-up:	3.0x		58.6x

Table 7: Time to obtain all maximal trap spaces. All times are in seconds and $|M|$ denotes the number of trap spaces.

	general		unitary	
Model name	$\|M\|$	$\texttt{mvn}^{\texttt{trap}}$	$\|M\|$	$\texttt{mvn}^{\texttt{trap}}$
BUDDING YEAST (146)	12	1.07	10	0.96
AGS CELL FATE (148)	11	0.04	11	0.04
TCR METABOLISM (151)	6	0.13	6	0.11
TH1-TH2 DIFFER. (155)	42	0.05	42	0.05
TH DIFFER. (157)	63	0.07	63	0.07
YEAST CORE (159)	10	0.58	8	0.57
IL17 DIFFER. (160)	30	0.07	30	0.07
MONOCYTES DIF. (161)	4	0.06	4	0.06
MESODERM SPEC. (167)	29	0.06	29	0.05
SEA URCHIN (175)	22	0.04	20	0.03
MYELOFIBR. ENV. (176)	7	0.04	7	0.04
MAST CELL (178)	8	0.03	8	0.03
MICROENV. CONTROL (179)	20	0.06	20	0.06
ALT. IN BLADDER (183)	8	0.04	8	0.04
BRAF TREATMENT (190)	10	0.03	10	0.03
SEGMENT POLARITY (192)	70	0.07	74	0.07
VULVAR PRECURSOR (194)	35	0.06	35	0.05
CTLA4 CHECKPOINT (195)	75	0.13	75	0.13
LYMPHOCYTE SPEC. (196)	10	0.07	10	0.07
ANTERIOR POST. (197)	9	0.09	8	0.08
MYC IN-VITRO	27	44.85	27	13.45
MYC IN-VIVO	21	45.32	21	13.43
METABOLISM	96	32.42	65	8.15
LEUKAEMIA	35	0.16	31	0.12
SKIN MODEL	78	0.24	62	0.18
VPC	38	0.09	34	0.08

Table 8: Time to compute the first fixed point. All times are in seconds. DNF denotes time-out of one hour. NM are non-monotonic networks which cannot be processed using MPBN.

	trap-mvn		trappist			
Model name	siphon	deadlock	siphon	deadlock	MPBN	AN-ASP
BUDDING YEAST (146)	0.94	0.53	2.03	1.10	DNF	0.76
AGS CELL FATE (148)	0.04	0.04	0.48	0.49	0.69	0.41
TCR METABOLISM (151)	0.10	0.09	0.63	0.60	9.85	0.47
TH1-TH2 DIFFER. (155)	0.05	0.04	0.48	0.48	NM	0.41
TH DIFFER. (157)	0.06	0.06	0.51	0.49	0.72	0.41
YEAST CORE (159)	0.57	0.41	1.43	0.90	DNF	0.64
IL17 DIFFER. (160)	0.06	0.06	0.51	0.50	0.74	0.45
MONOCYTES DIF. (161)	0.06	0.06	0.52	0.50	0.73	0.43
MESODERM SPEC. (167)	0.05	0.05	0.49	0.48	0.99	0.42
SEA URCHIN (175)	0.03	0.03	0.43	0.44	0.81	0.37
MYELOFIBR. ENV. (176)	0.04	0.04	0.46	0.45	0.72	0.38
MAST CELL (178)	0.03	0.03	0.43	0.43	0.63	0.37
MICROENV. CONTROL (179)	0.05	0.05	0.48	0.48	1.36	0.41
ALT. IN BLADDER (183)	0.04	0.04	0.44	0.44	0.66	0.38
BRAF TREATMENT (190)	0.03	0.03	0.45	0.45	0.65	0.38
SEGMENT POLARITY (192)	0.06	0.06	0.48	0.48	0.88	0.40
VULVAR PRECURSOR (194)	0.05	0.05	0.48	0.48	NM	0.40
CTLA4 CHECKPOINT (195)	0.09	0.08	0.57	0.60	NM	0.47
LYMPHOCYTE SPEC. (196)	0.07	0.06	0.48	0.48	NM	0.40
ANTERIOR POST. (197)	0.07	0.07	0.52	0.53	6.44	0.41
MYC IN-VITRO	13.24	4.31	18.42	4.77	DNF	2.95
MYC IN-VIVO	13.23	4.34	17.97	4.68	DNF	2.97
METABOLISM	7.84	4.57	7.33	3.57	NM	2.78
LEUKAEMIA	0.11	0.10	0.56	0.55	DNF	0.43
SKIN MODEL	0.15	0.13	0.79	0.72	4.76	0.48
VPC	0.08	0.07	0.52	0.54	1.15	0.40
